# Assigning Peptide Structure from Ion-Mobility Mass Spectrometry Collision Cross Section Data

**DOI:** 10.1021/jasms.5c00078

**Published:** 2025-07-07

**Authors:** Mithony Keng, Kenneth M. Merz

**Affiliations:** 1 Department of Chemistry, 3078 Michigan State University, East Lansing, Michigan 48824, United States; 2 Department of Biochemistry and Molecular Biology, 3078 Michigan State University, East Lansing, Michigan 48824, United States

## Abstract

The ion mobility (IM) technique coupled with traditional mass spectrometry (IM-MS) has introduced a practical tool for the characterization of peptide analyte ions by exploiting the difference in their collision cross section (CCS) values. CCS holds molecular level information that can be used to computationally assign the conformation(s) of a molecular system. However, a reliable and accurate method for peptide structure prediction remains a challenge because peptides exhibit dynamic gas-phase intramolecular interactions, and hence, the methods used will need to account for this. In this work, we systematically assessed the performance of a computational workflow involving both classical and density functional theory (DFT) steps to elucidate the peptide structure. Not unexpectedly, extensive enumeration of available peptide conformations was critical to obtain high-quality results. Due to the size of the systems studied and the large numbers of conformers that needed to be optimized, we initially chose the D3-B3LYP/6-31G­(d) level of theory and obtained good agreement between experimental and computed CCS values. However, in several cases, suboptimal accuracies were observed, but we found that increasing the basis set used to 6-31G­(d,p) was able to improve our agreement with experiment. Altogether, we demonstrated that accurate peptide structure assignment is achievable with adequate sampling of the conformational space and using the appropriate quantum mechanical level of theory to account for intramolecular interactions in the gas phase.

## Graphical abstract

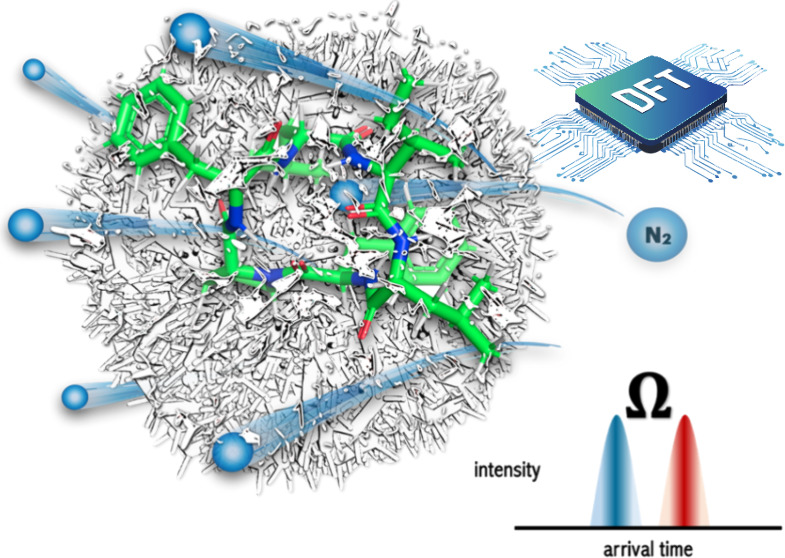

## Introduction

Trypsin digestion of a protein analyte in combination with mass spectrometry (MS) is one of the most common and practical methods for proteomic profiling of the metabolome. In most protein sequencing workflows, soft-ionization techniques such as matrix-assisted laser desorption/ionization and electrospray ionization (ESI) are utilized to generate precursor protein ions and fragment peptide ions,^1^ respectively, that are populated in unknown gas-phase conformations for MS analyses. The emergence of the ion mobility (IM) technique coupled with traditional MS (IM-MS) has added a practical quantitative and qualitative dimension to the characterization of biologically relevant peptide structures by exploiting the difference in the ions rotationally averaged collision cross section (CCS) values.^2,3^ This is realized because ions with larger conformations have a greater frequency of collision with neutral gases in the IM-MS drift region, resulting in differentiated drift velocities between analytes with identical *m*/*z*. Within the confines of IM-MS, the CCS can be derived from ion mobility (*K*) and thus is affected by both the ion drift velocity (*v*_d_) and the strength of the electric field, *E* (see eq 1).^4^ Depending on the IM technique, *E* can be applied as an electrodynamic field (e.g., TWIMS) or a uniform field (e.g., DTIMS).

$$K=\frac{v_{d}}{E}$$ 1

We note that there have been several computational works relevant to CCS measurements like using machine learning, molecular dynamics (MD), and other approaches to predict CCS values for peptides;^5–11^ however, the outcomes of these works did not result in any experimentally validated molecular geometry or a reliable high throughput method for gas-phase structure assignment. Although works by Kim et al. (2017) and Wu et al. (2023) were able to produce final gas-phase peptide structures, their investigations were limited to resolving one and two peptide species, respectively.^12,13^ Moreover, molecular modeling methods that rely solely on pairwise potentials for finding an equilibrium (ground state) peptide conformation are prone to mis-assigned structures.^14^

To advance proteomics and support unambiguous MS based structure assignment, it is essential to have reliable gas-phase peptide theoretical and experimental standards for evaluating experimental results or for the query of physiochemical properties. The development of a theoretical reference database is an ongoing challenge due to the richness of peptide conformations in the gas phase. Unlike carbohydrates or lipids, which are structurally rigid polymers or have minimal titratable sites, respectively, the conformational space of peptides is more expansive since they can be constructed from 20 common amino acids that have flexible side chains with different degrees of acidity/basicity. Consequently, this makes finding equilibrium charge sites on peptides for modeling IM-MS ions a major challenge and, accordingly, requires an accurate and efficient quantum mechanical theory to generate reliable gas-phase conformations.

Moreover, the challenge posed by peptide conformational dynamics in replicating experimental geometry was, for example, demonstrated by the interconversion of polyalanine from a 3_10_ helix to an α-helix conformation with increasing alanine residues using MP2/6-31G­(d) or the B97-D/def2-TZVP level of theory (interconversion onset began at alanine-8 for MP2 and at alanine-10 for B97-D).^15^ Importantly, when the B3LYP/6-311++G­(2d,2p) level with no long-range dispersion corrections was employed, the 3_10_ helix remained the more stable and, thus, the preferred conformation for alanine-4 through alanine-16. Based on these results for the different QM theories, it is inferred that a significant amount of the intramolecular interaction is attributable to the weakly attractive, but in aggregate significant, London dispersion force. Classically, the dispersion interaction is represented in the van der Waal (*E*_vdw_) energy component of the total noncovalent energy (*E*_nc_) of a system (eq 2). The energy attributed to nonbonded interaction is defined as

$$E_{\mathrm{nc}}=E_{\mathrm{vdw}}+E_{\mathrm{es}}$$ 2

*E*_es_ is the electrostatic term. The van der Waals energy and electrostatic energy for an interacting pair of atoms a and b are described by eq 3 and eq 4, respectively.^16^

$$E_{\mathrm{vdw}}=\sum \epsilon_{\mathrm{ab}}\left[{\left(\frac{r_{\min}}{r_{\mathrm{ab}}}\right)}^{12}-2{\left(\frac{r_{\min}}{r_{\mathrm{ab}}}\right)}^{6}\right]$$ 3

$$E_{\mathrm{es}}=\sum \frac{q_{a}q_{b}}{4\pi \varepsilon_{0}r_{\mathrm{ab}}}$$ 4

From our experience, for relatively small molecules where the contribution of nonbonded intramolecular interactions is very small, using the popular density functional theory (DFT) functional B3LYP with a small basis set such as 6-31G­(d) is often sufficient for obtaining reasonably accurate structures in a timely manner. However, when peptide ions are large enough that distant nonbonding atoms loop back and are sterically allowed to interact, the long-range intramolecular interactions can become a significant factor in stabilizing the preferred equilibrium geometry.

In this work, we assess and validate an efficient and reliable method for the structural assignment of gas-phase peptide ions from IM-MS experimental CCS data. For the electronic structure theory (e.g., QM calculation step), we have elected to use DFT for geometry optimization and single-point energy calculation, which has shown reasonably good generalizability in prior works^17–19^ for small molecule and biomolecule CCS modeling. Moving forward, to prevent confusion regarding the different DFT implementations, the labels D3, D3(0), and D3­(BJ) represent a general designation of the Grimme dispersion correction, the dispersion correction with the zero-damping scheme, and the dispersion correction with the Becke–Johnson damping scheme, respectively.^20^ Additionally, the workflow also involves the use of ensembles for peptide ions produced for charge modeling and extensive conformational sampling coupled with downstream CCS computation. Carrying out CCS computations will allow us to quantify our structure assignment accuracy by comparing our results with the available experimental IM-MS reference CCS data. A detailed layout of our workflow is shown in Figure 1.

**Figure 1:**
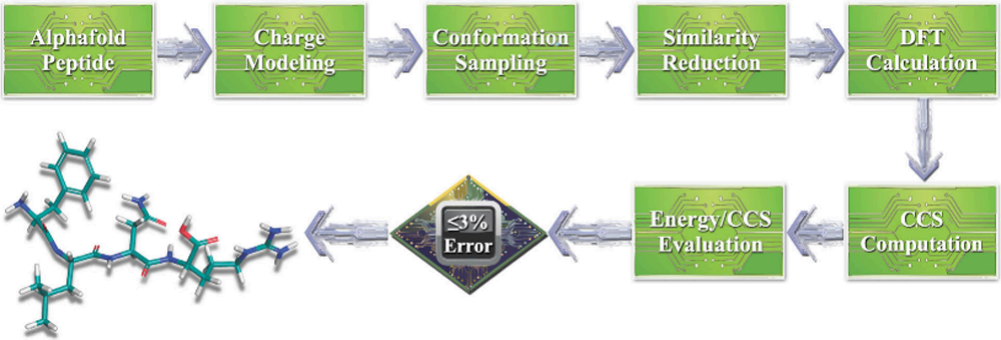
Schematic overview of the workflow for a successful designation of structure assignment for a gas-phase peptide ion from CCS data.

## Computational Methods

### Experimental CCS Values

The experimental CCS values for the 23 tryptic peptide systems with a range of 3–14 amino acids were retrieved from the Mclean research group Unified CCS Compendium database.^21,22^ Furthermore, the peptide analytes were produced via trypsin digestion and their arrival times (or drift time) were determined through a standardized interlaboratory protocol.^23^ To generate peptide ions in the [M + H]^+^ charge mode, electrospray ionization (ESI) was used as the ionization method. The averaged drift times from IM-MS experiments were used to calculate the CCS values through the Mason–Schamp relationship for low-field ion mobility (eq 5),

$$\Omega =\frac{{\left(18\pi \right)}^{1/2}}{16}\frac{ze}{{\left(k_{b}T\right)}^{1/2}}{\left[\frac{1}{m_{i}}+\frac{1}{m_{b}}\right]}^{1/2}\frac{t_{A}E}{L}\frac{760}{P}\frac{T}{273.15}\frac{1}{N}$$ 5

where *z* is the ion charge, *k*_b_ is the Boltzmann constant, *T* is the temperature of the drift tube, *e* is the electron charge, *m*_i_ is the ion mass, *m*_b_ is the buffer gas mass, *t*_A_ is the arrival time of the ion analyte, *E* is the electric field strength, *P* is the drift tube pressure, and *N* is the number density of the buffer gas. For this work, N_2_ is used as the drift gas; therefore, only the data and results related to N_2_ are considered.

### Peptide Seed Generation

The peptide starting structures were generated using the protein structure prediction software^24^ AlphaFold2. To prevent structural violations or clashes, the Amber99SB force field with harmonic constraints was used to minimize and relax the predicted peptide structures. Other parameters that were used in the AlphaFold2 structure generation step were the “MMseq2­(UniRef+Enivoronmental)” option for the msa mode; “unpaired+paired” option for the pair mode; the model type was set at auto; and the number of recycles was set at 3. The AlphaFold2 run for each peptide query sequence produced five model conformations with a structure prediction quality grading for model one being the most accurate and model five being the least. Moreover, for all the peptide systems predicted by AlphaFold2 only the model one structure was chosen as the seed for the conformation generation step.

### Conformation Generation

From the seed structure, models representing unique protonated +1 charge (i.e., [M + H]^+^) peptide species were generated according to their protonation behaviors with the aid of Maestro^25^ p*K*_a_ prediction program from Schrodinger. Next, 1000 conformers were then generated from the parent protomer model utilizing the Schrodinger conformation generation software ConfGen.^26,27^ We chose not to enable the OPLS3 force-field option for structure minimization because this would significantly reduce the number and diversity of conformations generated, which would have limited the amount of conformational space sampled. The steps that followed utilized the workflow established in our previous work^19^ on QM aided CCS calculation for metabolite annotation.

### Conformation Optimization and Structure Similarity Reduction

Next, the 1000 conformers of each peptide protomer model were preoptimized with the neural network potential ANI.^28^ The resultant peptide conformers were clustered into ensembles of peptides with similar conformations (as measured by RMSD values), and from each ensemble, we extracted the centroid peptide (as a representative of the ensemble) for further processing. This was accomplished using the open-source program Autograph.^29^ Generally, the number of initial conformers per peptide was reduced to 14–25 conformer centers after clustering. The Autograph step is necessary to reduce the computational workload in the QM optimization step.

### QM Geometry Optimization, Single-Point Energy, and Charge Calculations

The QM processing for the ensembles was accomplished using the Gaussian16 software^30^ package. For geometry optimization of the conformer centers and the subsequent single-point energy and (Mulliken) charge calculation steps, we utilized the DFT hybrid-GGA functional B3LYP with Pople’s split-valence basis set of 6-31G­(d). Additionally, to better account for contributions from dispersion effects on the ground state geometry, dispersion corrections using the D3 variants D3(0) and D3­(BJ) with this DFT level of theory were applied to the same conformer centers produced from Autograph in two independent runs (one for each dispersion method).

Additionally, to improve the representation of the gas-phase geometry, the 6-31G­(d) basis set was expanded to 6-31G (d,p) to include a polarization function for all hydrogen atoms. We again used the B3LYP functional. The protonation state that initially produced the energy minimum candidate conformation(s) at the D3-B3LYP/6-31G­(d) level of theory was carried forward to the D3-B3LYP/6-31G­(d,p) level of theory. Again, we employed both D3 variants in independent runs. The *XYZ* files containing the centers produced from the initial Autograph clustering (raw ensemble) step were recycled and used as the starting structure for this run. The ensuing steps used to produce this batch of CCS values followed the same workflow and software parameters as those used in the initial run. Finally, the D3 variant (D3(0) or D3­(BJ)) that produced a lower CCS error was then used to select the new candidate conformation with the lowest relative energy.

### Collision Cross Section Calculation

The CCS values were calculated using the peptide DFT optimized geometry and corresponding partial charge value with the aid of the open-source software High Performance Collision Cross Section^31^ (HPCCS). This software uses the trajectory method, which requires solving both an orientationally averaged collision integral and an intermolecular interaction potential between the ion analyte and the buffer gas. The HPCCS configuration parameters were set to 1 for the number of conformations per input, 10 for average mobility cycles, 20 for velocity integration, 500 for Monte Carlos integrations, 1000 for the number of rotations in average potential, 298.0 for temperature (kelvin), and 2 for N_2_ buffer gas. The final computed CCS value for an ensemble is a Boltzmann weighted average CCS using the relative electronic energies of conformers that are within ≤3 kcal mol^–1^ of the energy minimum conformer. The final peptide structures were visually inspected using PyMol.^32^

To be consistent with our previous works^19^ on CCS, we judged the accuracy performance of our prediction by using a 3% error threshold between the experimental and computed CCS values. This threshold allows us to account for the uncertainty that is historically encountered from experimental CCS values calculated using the Mason–Schamp equation and for laboratory IM-MS instrument calibration error.^33,34^

## Results and Discussion

### Structure Prediction Performance of B3LYP/6-31G­(d) and D3-B3LYP/6-31G­(d)

As stated previously, we started the DFT calculation step in our workflow using the smaller 6-31­(d) basis set initially and proceeded accordingly in response to how reliable this level of theory was in assigning experimentally viable structures. This helped us to explicitly determine whether a larger basis set with auxiliary functions (hence greater computational cost) is necessary to produce successful gas-phase structure assignment. Detailed results containing the ensemble relative energetics and CCS prediction performance for the 23 test peptide ions using B3LYP/6-31G­(d), D3(0)-B3LYP/6-31G­(d), and D3­(BJ)-B3LYP/6-31G­(d) are tabulated in Tables S1–S23 in the Supporting Information.

With the implementation of the smallest DFT level of theory for this work (i.e., B3LYP/6-31G­(d)), we obtained a structure prediction accuracy success rate of ∼22% (5 out of 23 systems achieve ≤3% CCS error) with an average CCS error of ∼9% at a standard deviation of ±6% (Table 1). We observe that the 18 peptide systems with suboptimal predictions (i.e., >3% error) all have computed CCS values that overestimate experiment, which translates to predicted ground state structures that exhibited a more extended conformation, and for isolated molecular ions in the gas-phase, this hints that the intramolecular interactions were underestimated during the DFT geometry optimization process.

**Table 1:** Computed Boltzmann-Weighted CCS Values for Singly Protonated, [M + H]^+^, Peptide Structures Processed at the DFT B3LYP/6-31G­(d) Level of Theory with Different Dispersion Models

	peptide ions	exp CCS (Å^2^)	B3LYP/6-31G(d) CCS (Å^2^)	D3(0)-B3LYP/6-31G(d) CCS (Å^2^)	D3(BJ)-B3LYP/6-31G(d) CCS (Å^2^)
1	ANELLINVK	322.50	347.62 (7.8)	322.93 (0.1)	342.93 (6.3)
2	AWEVTVK	278.20	333.91 (20.1)	295.78 (6.3)	296.11 (6.4)
3	AWSVAR	255.70	290.00 (11.8)	264.29 (3.4)	274.98 (7.5)
4	DYYFALAHTVR	374.80	410.42 (9.5)	412.60 (10.1)	422.64 (12.8)
5	ELR	200.30	197.81 (1.2)	203.43 (1.6)	201.62 (0.7)
6	EWTR	229.70	250.20 (8.9)	241.93 (5.3)	243.40 (6.0)
7	EYK	203.20	213.81 (5.2)	210.64 (3.7)	209.78 (3.2)
8	FAAYLER	293.10	335.07 (14.3)	302.10 (3.1)	307.17 (4.8)
9	FLNR	231.60	250.13 (8.0)	231.08 (0.2)	238.48 (3.0)
10	FPK	184.70	193.09 (4.5)	188.53 (2.1)	188.59 (2.1)
11	FSSDR	234.50	250.05 (6.6)	250.86 (7.0)	240.25 (2.5)
12	GLVK	205.30	210.80 (2.7)	210.69 (2.6)	210.63 (2.6)
13	LWSAK	237.70	267.85 (12.7)	243.40 (2.4)	243.63 (2.5)
14	NFNR	224.00	252.51 (12.7)	247.61 (10.5)	239.92 (7.1)
15	NIATGSK	255.30	298.74 (17.0)	257.95 (1.0)	248.67 (2.6)
16	poly-6-glycine	171.04	176.67 (3.3)	174.29 (1.9)	170.03 (0.6)
17	poly-8-glycine	206.52	203.34 (1.5)	201.43 (2.5)	197.56 (4.3)
18	poly-10-glycine	216.15	232.7 (7.7)	222.58 (3.0)	222.02 (2.7)
19	poly-14-glycine	254.51	277.82 (9.2)	266.42 (4.7)	266.33 (4.6)
20	TFAEALR	281.80	339.29 (20.4)	304.43 (8.0)	295.93 (5.0)
21	TIAQYAR	280.00	315.95 (12.8)	282.10 (0.8)	300.94 (7.5)
22	VASLR	232.00	230.65 (0.6)	223.39 (3.7)	232.05 (0.0)
23	WIR	213.20	210.41 (1.3)	217.68 (2.1)	208.50 (2.2)
average % error^a^			8.8 ± 6	3.7 ± 3	4.2 ± 3

a The average of values in parentheses.

Although energetics obtained at the MP2 level using HF/6-31­(d,p) geometries showed good agreement with experiment for small peptides, extending this to peptides of the size studied herein is still computationally intensive.^35^ With that consideration, we added a computationally inexpensive empirical dispersion correction to the initial B3LYP/6-31G­(d) level of theory for geometry optimization, single-point energy calculation, and charge calculation. Since there is no available metric or consensus in the computational chemistry community on which damping function for the D3 dispersion correction works best for peptides, we used both D3(0) and D3­(BJ) independently, and thus, we also report in this manuscript the effect of a damping function choice on the quality of structure prediction for peptide ions. Even though D3(0) and D3­(BJ) give rise to more repulsion and attraction at short-range interatomic distances,^36^ respectively, on the potential energy surface, there is no trend where one damping function favors a smaller or larger peptide conformation over another. Overall, we found that accounting for long-range dispersion reduces the average CCS error to ∼4% ± 3 (success rates of ∼52% for D3(0) and ∼47% for D3­(BJ)), which indicates a significant improvement in both the accuracy and transferability of our method.

Additionally, we measured the distance between our reference points N-terminal amine and the C-terminal carboxylic acid Cα for the peptide ions since it has been previously shown that conformation compactness is decreased with side chain–side chain interactions.^37–39^ This is especially relevant here since most of the charge sites assigned to the test systems are located on the terminal residues (for the location of assigned amino acid charge site(s), see Figure S1). Thus, this measurement provides a good quantitative indication of structural compactness since the distance between N to C-terminal is related to the packing efficiency of amino acid residues, driven by intramolecular interactions. For the 23 candidate peptides measured, the average N to C-terminal distance is ∼7 Å with dispersion correction compared to ∼8.7 Å without dispersion (Figure 2A). Moreover, Figure 2B shows an example of the DFT theory dependent major and minor shifts in the N to C distance for the equilibrium structures of AWSVAR and FSSDR that correlate well with their computed CCS values, which we can attribute to the overall packing efficiency of the amino acids. It should be remarked that generally our computed CCS values for the peptide systems overestimate the reference CCS; therefore we believe that equilibrium structures that are assigned using the competing DFT levels of theory that yield the smaller N to C-terminal will on average have better agreement with experiment. When we averaged the CCS values for the D3(0) and D3­(BJ), the difference in N to C-terminal distance translates to a ∼7.5% and ∼1.8% larger CCS values for the dispersion uncorrected equilibrium conformations for AWSVAR and FSSDR, respectively.

**Figure 2:**
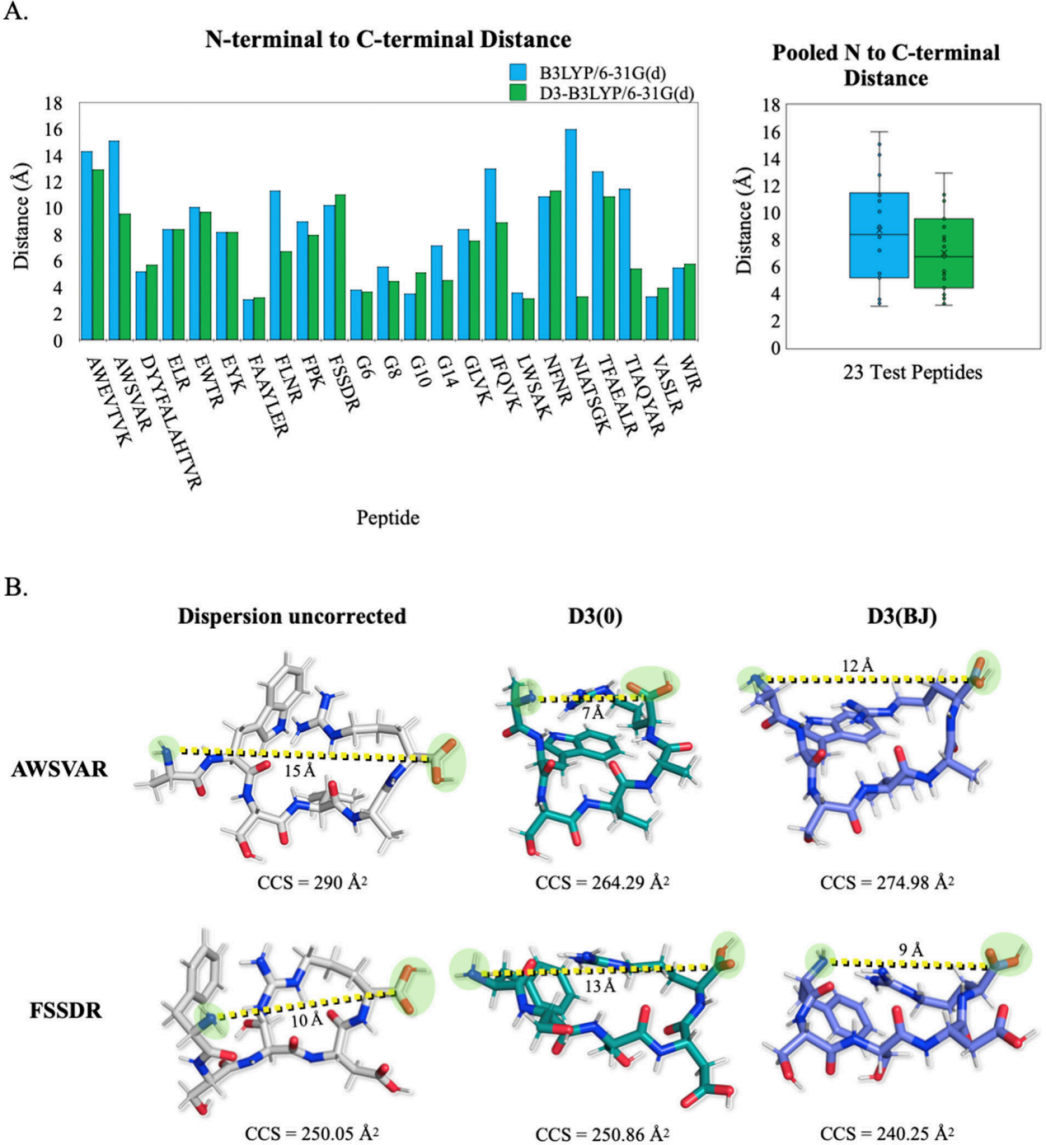
(A) Individual and pooled average N to C-terminal distances for 23 peptide ion equilibrium structures at different levels of DFT theories. The two distinctions here are geometry optimization with unsupported (blue bar) and supported (green bar) long-range dispersion. (B) N to C-terminal (green highlight group) distance measurements (yellow dash) and computed CCS values for AWSVAR and FSSDR at three different DFT dispersion statuses.

### Addition of a *p*-Type Polarization Function

The residual overestimation of experimental CCS using D3-B3LYP/6-31G­(d) is evidence that further improvement in the level of theory might have some benefits. Much of the main chain and side chain of peptides have hydrogen bond donors and/or acceptors, and their propensity to form hydrogen bond interactions require that polar contacts be ideally within a distance of approximately 2.8 Å.^40,41^ Moreover, the B3LYP functional with the D95­(d,p) augmented basis set was routinely used in numerous works by Dannenberg and co-workers for modeling peptide in the gas-phase.^14,42–45^ Thus, following their well-established precedent, we added an additional *p*-type polarization function for hydrogen atoms (i.e., 6-31G­(d,p)) with the aim to improve upon our initial results. Although previous works^38,46^ comparing 6-31G­(d) and 6-31G­(d,p) have revealed negligible differences in resulting peptide conformation energetics (i.e., *cis*–*trans* conversion barrier) and geometric parameters (i.e., bond length and torsion angle), we found that the noted differences in the atomic charges produced in these works was worth pursuing further as a way to improve structure assignment success.^47^ As already mentioned, Mulliken atomic partial charges are used in HPCCS calculations employing the trajectory method, which makes these calculations sensitive to atomic charges. For example, a survey (Figure 3A) of atomic charges for the peptide ELR shows a subtle difference from atom to atom between the equilibrium conformations assigned by 6-31G­(d) and 6-31­(d,p). Nevertheless, in concert with the partial charge changes and their effect on CCS calculations, we obtained slightly more compact conformations for 6-31­(d,p) that better align with experimental data (see Figure 3B,C).

**Figure 3:**
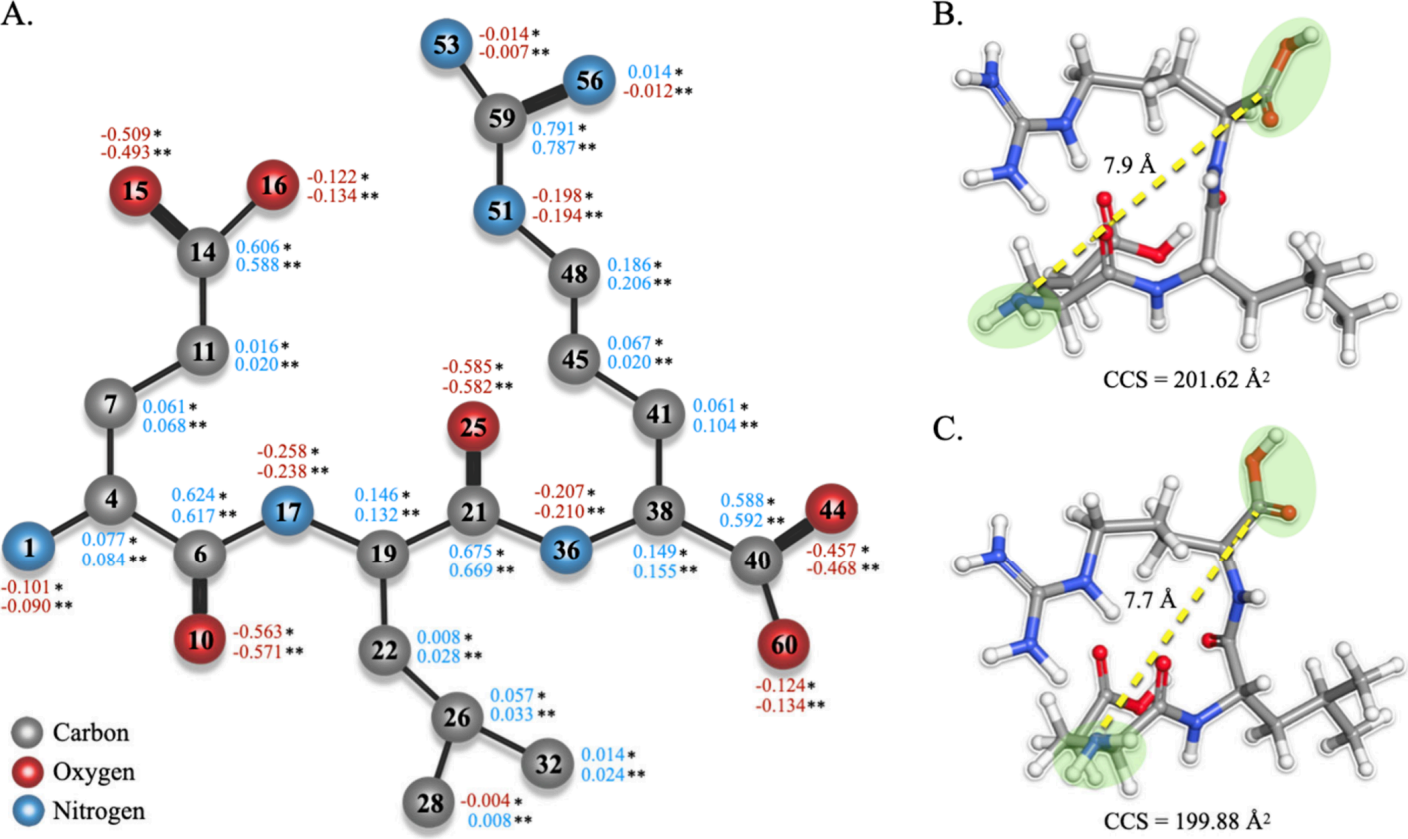
Effect of *p*-type polarization function on the ground-state electronic peptide structure. (A) ELR, [M + H]^+^, ion atomic partial charge distribution for heavy atoms (hydrogen summed) using the Mulliken charge scheme for 6-31G­(d) and 6-31G­(d,p). The value in red and blue indicates negative and positive partial charge, respectively; 6-31G­(d) and 6-31G­(d,p) are represented on the right of partial charge values by * and **, respectively. (B) N to C-terminal distance and computed CCS values for equilibrium ELR conformation obtained from 6-31G­(d) and (C) 6-31G­(d,p) basis sets. Both conformations are protonated at the same atomic site.

To further probe the significance of the choice of atomic point charges, we recomputed the CCS for eight arbitrarily selected peptides (ranging from 3 to 9 residues) using atomic partial charges calculated with D3BJ-B3LYP/6-31G­(d) on D3BJ-B3LYP/6-31G­(d,p) optimized geometries (i.e., D3BJ-B3LYP/6-31G­(d)//D3BJ-B3LYP/6-31G­(d,p)). Since the geometries between the two computed CCS values are identical, any difference in their CCS is entirely due to their respective partial charge distributions. As Figure 4 shows, CCS calculated using 6-31G­(d,p) charges is significantly larger than for 6-31­(d) for large peptides (i.e., ANELLINVK, AWEVTVK, TFAEALR). We hypothesize that this is because 6-31G­(d,p) produces a greater partial charge separation. For a given ion, this ultimately resulted in a stronger dipole-induced dipole effect on the colliding N_2_ buffer gas and therefore a decreased in ion mobility (stronger ion–neutral interaction). On the other hand, 6-31G­(d) significantly underestimated CCS values for the D3BJ-B3LYP/6-31G­(d,p) optimized geometries for the same large peptides. For the tested peptides, we found that the CCS changes with the 6-31G­(d,p) partial charges are favorable for achieving a much better agreement with experiment.

**Figure 4:**
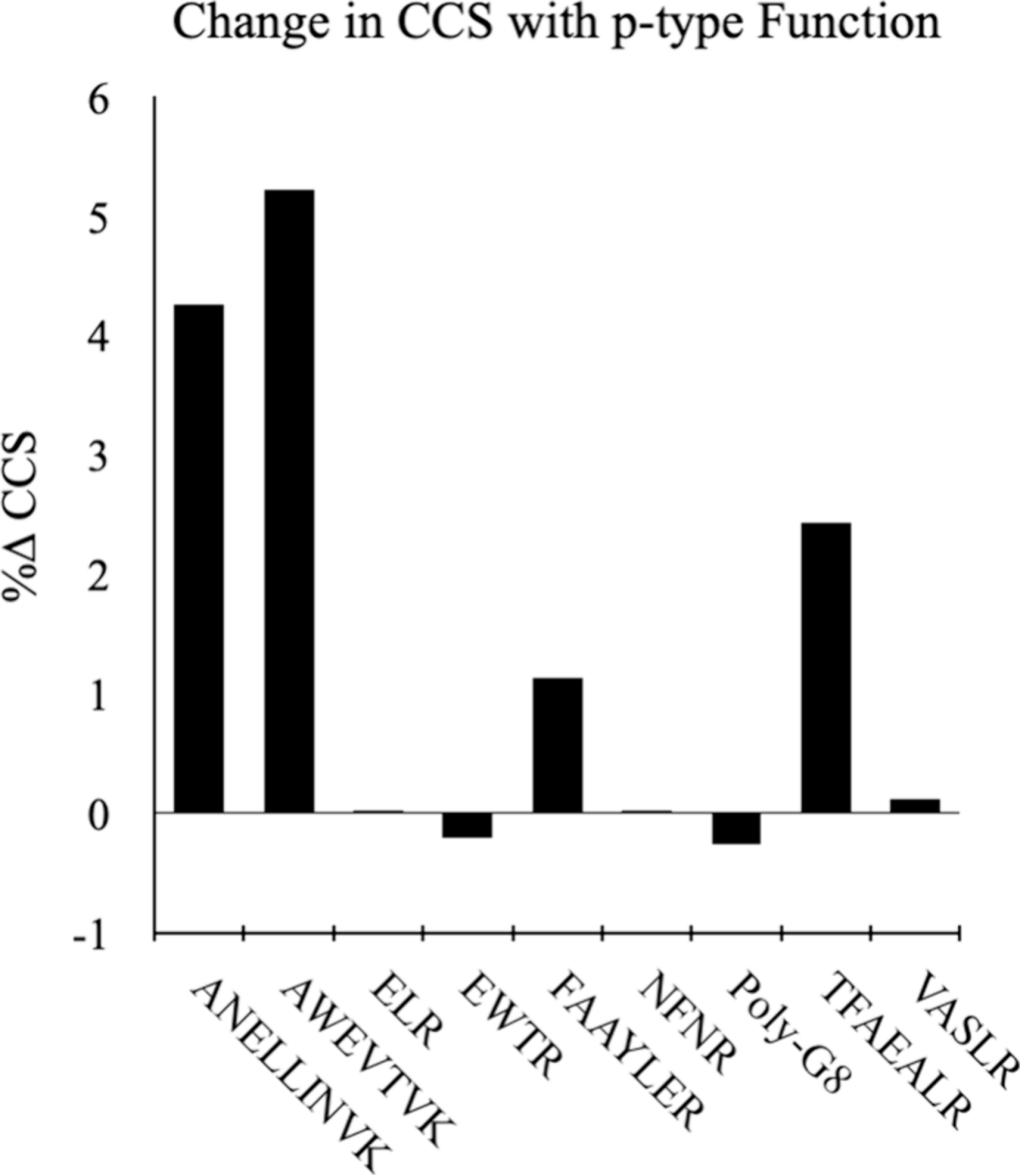
Percent change in CCS going from partial charges calculated with 6-31G­(d) to the 6-31­(d,p) basis set while maintaining the 6-31­(d,p) geometry. The DFT levels of theory for charge calculation are D3BJ-B3LYP/6-31G­(d)//D3BJ-B3LYP/6-31G­(d,p).

The complete performance results for the assigned equilibrium structures generated with this larger basis set are displayed in Table 2 (for DFT results at the conformer level per ensemble charge model, see Tables S24–S46 in the Supporting Information). The addition of the *p*-type function significantly increases the prediction success rate to ∼74% (17 out of 23 peptide systems achieved ≤3% CCS error) at a lower average CCS error of ∼2.2% ± 1.3. Furthermore, the average CCS overestimation for D3-B3LYP/6-31G­(d,p) is ∼1% ± 2.4 compared to 2% for D3-B3LYP/6-31G­(d). It should be noted that the lower standard deviation achieved with this level of theory increases the transferability of this method to larger peptides or even other biomolecule classes. Given the average N to C-terminal distance measurements shown in Figure 5, which shows only an average marginal difference (∼0.2 Å), we believe that the general improved accuracy in structure assignment at this point is still a combination of a more precise description of both intramolecular and intermolecular effects.

**Table 2:** Computed Boltzmann-Weighted CCS Values for Singly Protonated, [M + H]^+^, Peptide Structures Processed at the DFT B3LYP/6-31G­(d,p) Level of Theory with Different Dispersion Models

	peptide ions	D3(0)-B3LYP/6 31G(d,p) CCS (Å^2^)	D3(BJ)-B3LYP/6-31G(d,p) CCS (Å^2^)	D3-B3LYP/6-31G(d,p) CCS (Å^2^)^b^
1	ANELLINVK	335.91 (4.2)	332.21 (3.0)	332.21 (3.0)
2	AWEVTVK	286.04 (2.8)	294.42 (5.8)	286.04 (2.8)
3	AWSVAR	274.57 (7.4)	265.11 (3.7)	265.11 (3.7)
4	DYYFALAHTVR	389.49 (3.9)	392.12 (4.6)	389.49 (3.9)
5	ELR	207.93 (3.8)	199.88 (0.2)	199.88 (0.2)
6	EWTR	237.44 (3.4)	237.03 (3.2)	237.03 (3.2)
7	EYK	209.74 (3.2)	209.02 (2.9)	209.02 (2.9)
8	FAAYLER	292.77 (0.1)	287.53 (1.9)	292.77 (0.1)
9	FLNR	237.89 (2.7)	237.60 (2.6)	237.60 (2.6)
10	FPK	188.09 (1.8)	187.84 (1.7)	187.84 (1.7)
11	FSSDR	241.62 (3.0)	241.31 (2.9)	241.31 (2.9)
12	GLVK	208.71 (1.7)	211.01 (2.8)	208.71 (1.7)
13	LWSAK	236.19 (0.6)	250.71 (5.5)	236.19 (0.6)
14	NFNR	219.20 (2.1)	217.09 (3.1)	219.20 (2.1)
15	NIATGSK	260.08 (1.9)	253.95 (0.5)	253.95 (0.5)
16	Poly-6-glycine	172.39 (0.8)	175.29 (2.5)	172.39 (0.8)
17	Poly-8-glycine	197.76 (4.2)	197.50 (4.4)	197.76 (4.2)
18	Poly-10-glycine	225.89 (4.5)	219.44 (1.5)	219.44 (1.5)
19	Poly-14-glycine	264.76 (4.0)	263.74 (3.6)	263.74 (3.6)
20	TFAEALR	295.01 (4.7)	290.69 (3.2)	290.69 (3.2)
21	TIAQYAR	280.33 (0.1)	277.79 (0.8)	280.33 (0.1)
22	VASLR	228.70 (1.4)	228.3 (1.6)	228.70 (1.4)
23	WIR	205.63 (3.6)	210.41 (1.3)	210.41 (1.3)
average % error^a^		2.98 ± 1.8	2.75 ± 1.5	2.19 ± 1.3

a The average of the values in parentheses.

b Column contains CCS values from D3(0) or D3­(BJ) method that better agree with experiment.

**Figure 5:**
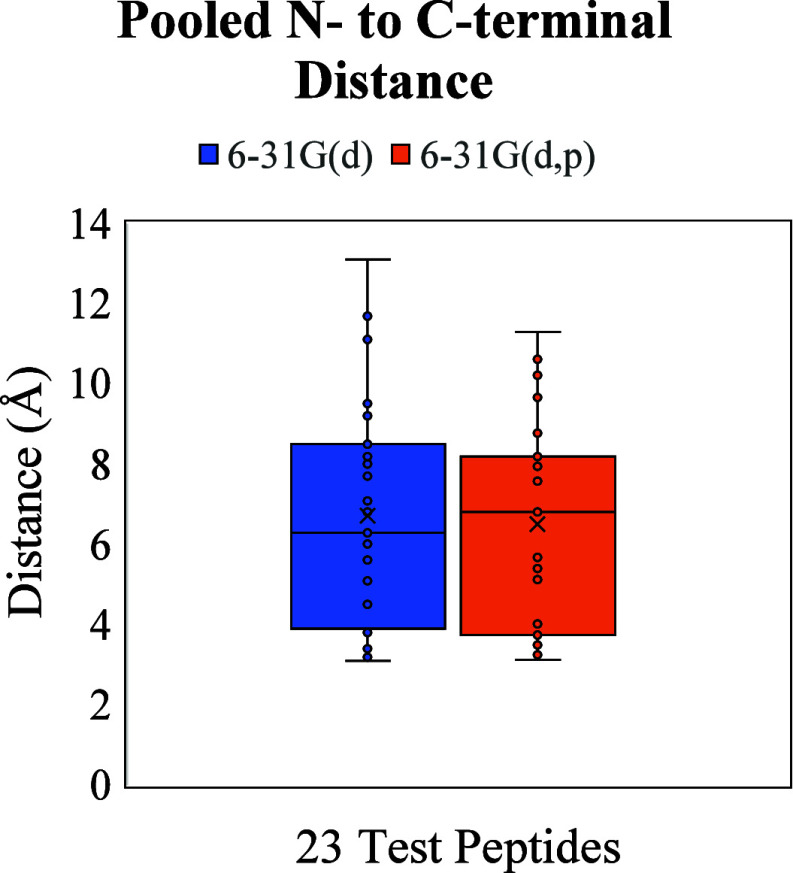
Pooled measurements of N to C-terminal distances for 23 peptides at the DFT D3-B3LYP level with 6-31G­(d) basis set (blue) or 6-31G­(d,p) basis set (orange). Average change in distance of (d ) → (d,p) is ≈−0.2 Å.

While the inclusion of dispersion^48,49^ and polarization functions^47^ have been noted to improve peptide equilibrium geometry optimization in the past, this work is notable for showing the significant effect on the CCS of gas-phase peptides. Figure 6A summarizes the CCS response to the different levels of theory, which again reinforces the need to account for long-range dispersion for sizable molecular systems. That said, the performance of the dispersion correction with different damping function can vary on a system basis; however, we found that increasing the basis set size (i.e., 6-31G­(d) → 6-31G­(d,p)) can reduce the disparity between D3(0) and D3­(BJ) CCS values (Figure 6B).

**Figure 6:**
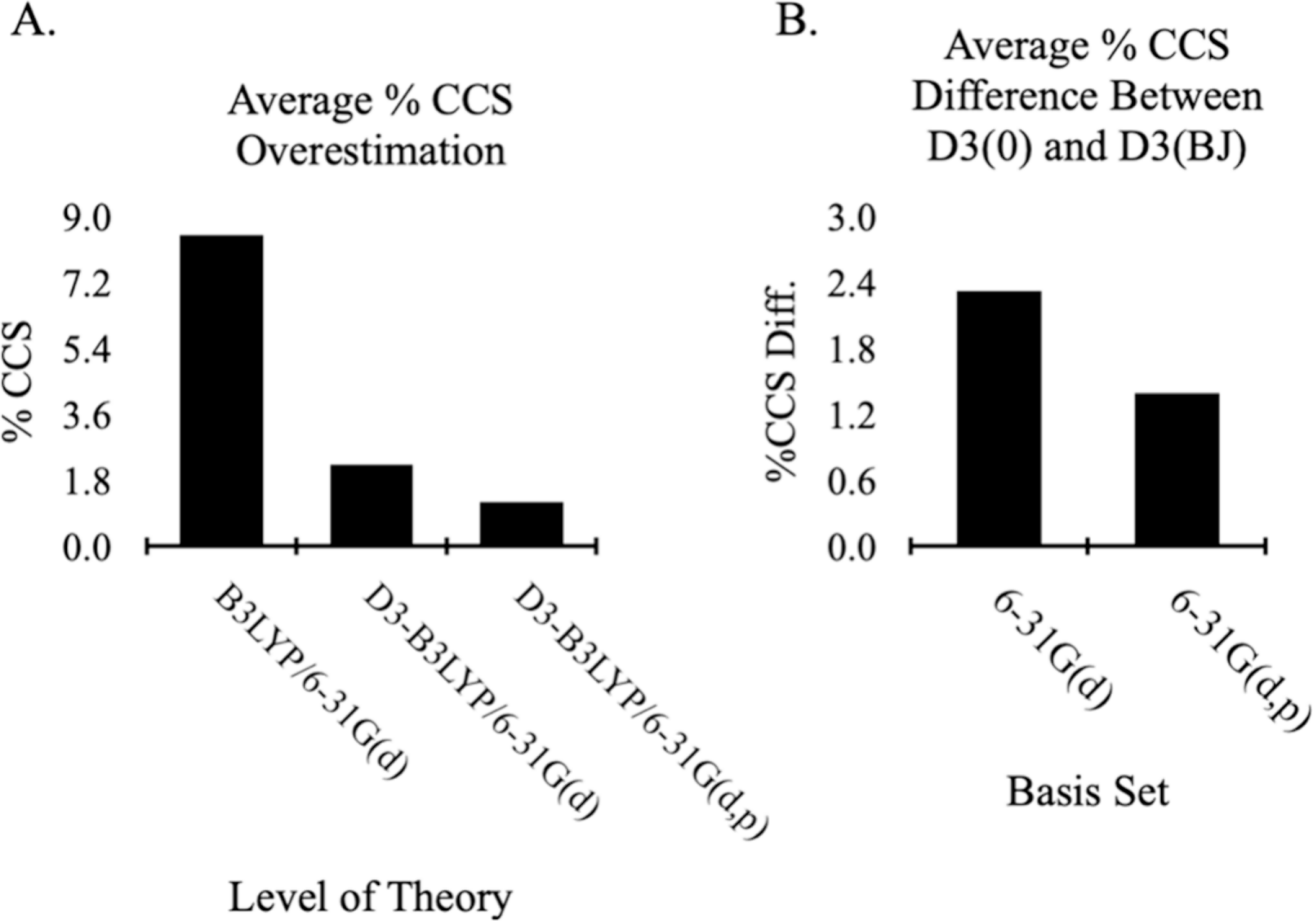
Trend for (A) average % CCS overestimation at three different DFT level implementations and for (B) effect of basis set auxiliary function on absolute % CCS difference between two dispersion correction methods.

## Conclusion

When peptide analytes in solution are converted and transferred to gas-phase ions for IM-MS analysis, there are intramolecular interactions that actively and significantly contribute to the equilibrium geometry. In this work, we have established a practical method that utilizes extensive conformational modeling coupled with CCS computation to account for and quantify gas-phase intramolecular interactions for the accurate elucidation of peptide ion structures. For the DFT geometry optimization step, we demonstrated that the B3LYP/6-31G­(d) level of theory with dispersion correction significantly improved the agreement between experimental and calculated CCS values. However, the performance for this DFT level of theory is still unsatisfactory, since only marginally half of the predicted structures generated achieved an acceptable CCS error below the limit set for this work. Accordingly, we found that increasing the basis set used to 6-31G­(d,p) substantially improved accuracy, success rate, and transferability of our structure assignment method by better recovering the ion–neutral collision interaction. Altogether, we have systematically demonstrated that significant improvement in structure elucidation for peptide ions of the [M + H]^+^ type of 3–14 amino acids is achievable with the appropriate quantum mechanical level of theory that accounts for intramolecular and intermolecular interactions in the gas phase.

## Supplementary Material



## Notes

Peptide ensemble DFT energies per dispersion status, assigned peptide amino acid charge site(s), and peptide ensemble DFT energies per basis set type (PDF)
