# Supplementary material for: Assigning Peptide Structure from Ion-Mobility Mass Spectrometry Collision Cross Section Data
Source: J Am Soc Mass Spectrom. 2025 Jul 7;36(8):1677–85. doi: 10.1021/jasms.5c00078 (PMC12333332; doi:10.1021/jasms.5c00078)
Supplement: Supplementary file 1 [file js5c00078_si_001.pdf]

Supporting Information

Assigning Peptide Structure from Ion-Mobility Mass Spectrometry Collision Cross Section Data

Mithony Keng and Kenneth M Merz, Jr.*

Department of Chemistry, Michigan State University,

East Lansing, Michigan 48824, United States

Department of Biochemistry and Molecular Biology, Michigan State University,

East Lansing, Michigan 48824, United States

*Corresponding Author: Kenneth M. Merz

*Corresponding Author Email: merz@chemistry.msu.edu

Table S1-S23 contain ensembles for assigned peptide protomers, [M+H]+, that have been processed for geometry optimization and single-point energy calculation at the DFT B3LYP/6-31G(d) level of theory without or with two different dispersion correction methods (*i.e.*, D3(0) and D3(BJ).

Figure S1 shows in single-letter amino acid code form where charge resides on the peptide for each assigned structure per [M+H]+ system.

Table S24-S46 contain ensembles for assigned peptide protomers, [M+H]+, that have been processed for geometry optimization and single-point energy calculation at the DFT B3LYP/6-31G(d,p) level of theory without or with two different dispersion correction methods (*i.e.*, D3(0) and D3(BJ).

Table S1. ANELLINVK Candidate Structures CCS Performance for 6-31G(d)

Dispersion Uncorrected				
Conformation No.	Model	Relative Energy (kcal/mol)	Mol Fraction	CCS % Error
112	1	79.88	0	25.57
234	1	74.19	0	26.21

472	1	78.31	0	26.09
474	1	39.20	0	23.80
535	1	32.59	0	26.87
549	1	0	1	7.23
584	1	64.96	0	23.46
607	1	50.36	0	26.80
684	1	75.21	0	28.31
727	1	43.65	0	28.31
966	1	40.55	0	28.41
D3(0) Dispersion Corrected				
102	1	25.26	0	16.60
137	1	0.00	1	0.13
234	1	39.96	0	14.14
367	1	8.72	0	14.31
879	1	57.33	0	18.52
D3(BJ) Dispersion Corrected				
102	1	11.15	0	13.81
137	1	13.66	0	0.93
234	1	0.00	1	5.96
367	1	7.68	0	15.98
879	1	43.03	0	19.32

Table S2. AWEVTVK Candidate Structures CCS Performance for 6-31G(d)

Dispersion Uncorrected				
Conformation No.	Model	Relative Energy (kcal/mol)	Mol Fraction	CCS % Error
34	2	16.99	0	11.92
102	2	14.36	0	19.59
231	2	0.00	1	16.69
472	2	16.38	0	18.17
731	2	51.29	0	21.15
772	2	12.35	0	15.56
879	2	24.54	0	18.84
D3(0) Dispersion Corrected				
34	2	11.64	0	12.80
102	2	22.62	0	15.65
231	2	27.13	0	17.92
234	2	27.15	0	15.18
472	2	23.00	0	14.14
731	2	51.62	0	16.27
772	2	0.00	1	5.94
879	2	35.55	0	17.56
D3(BJ) Dispersion Corrected				
34	2	27.46	0	13.28
102	2	46.01	0	19.82

234	2	36.53	0	17.04
472	2	3.94	0	9.76
731	2	62.79	0	16.16
772	2	0.00	1	6.04
879	2	31.96	0	15.75

Table S3. AWSVAR Candidate Structures CCS Performance for 6-31G(d)

Dispersion Uncorrected				
Conformation No.	Model	Relative Energy (kcal/mol)	Mol Fraction	CCS % Error
34	2	31.36	0	22.19
102	2	21.22	0	20.47
121	2	3.48	0	14.75
126	2	0	1	11.82
197	2	16.66	0	18.05
217	2	18.69	0	17.6
239	2	11.92	0	15.45
418	2	16.58	0	16.9
448	2	17.27	0	17.5
472	2	8.19	0	16.71
474	2	9.57	0	11.45
496	2	29.91	0	21.67
549	2	18.68	0	21.15
662	2	14.87	0	17.23
669	2	23.06	0	17.06
684	2	24.85	0	18.84
727	2	22.13	0	19.63
879	2	33.38	0	23.73
961	2	10.59	0	14.22
962	2	40.72	0	27.9
966	2	8.61	0	12.88
D3(0) Dispersion Corrected				
962	2	0	0.96	3.07
727	2	1.86	0.04	7.29
966	2	9.36	0	11
239	2	10.09	0	10.67
121	2	11.53	0	10.98
474	2	13.06	0	6.94
472	2	13.31	0	14.8
961	2	14.58	0	9.15
197	2	14.61	0	11.82
669	2	17.87	0	15.24
448	2	19.11	0	14.21
126	2	19.64	0	10.61
217	2	21.1	0	15.95

418	2	25.89	0	19.33
662	2	27.37	0	17.49
684	2	28.27	0	13.77
102	2	29.14	0	6.79
549	2	32.79	0	21.57
879	2	41.2	0	21.46
496	2	41.55	0	18.7
34	2	46.87	0	17.68
D3(BJ) Dispersion Corrected				
727	2	0	1	7
662	2	5.67	0	9.09
966	2	6.85	0	10.98
121	2	9.39	0	10.55
472	2	10.81	0	14.63
197	2	11.73	0	11.79
961	2	12.17	0	9.23
239	2	12.66	0	14.41
669	2	15.1	0	15.01
962	2	16.54	0	1.91
418	2	16.91	0	15.24
126	2	16.98	0	10.67
217	2	18.64	0	15.56
448	2	21.87	0	15.15
684	2	25.68	0	13.46
496	2	27.16	0	18.31
474	2	28.27	0	7.35
549	2	29.78	0	21
102	2	31.79	0	9.73
879	2	38.26	0	21.7
34	2	44.33	0	17.56

Table S4. DYYFALAHTVR Candidate Structures CCS Performance for 6-31G(d)

Dispersion Uncorrected				
Conformation No.	Model	Relative Energy (kcal/mol)	Mol Fraction	CCS % Error
2	2	12.80	0	27.13
95	2	31.07	0	19.85
156	2	46.23	0	32.43
232	2	28.03	0	29.88
306	2	95.01	0	31.54
535	2	50.09	0	30.65
617	2	49.33	0	30.02
647	2	56.24	0	25.66
839	2	0.00	1	8.68
997	2	45.18	0	22.93
D3(0) Dispersion Corrected				

2	2	74.63	0	29.79
95	2	1.64	0.06	4.38
156	2	64.39	0	27.11
232	2	37.91	0	14.23
306	2	64.89	0	27.45
535	2	59.56	0	21.47
617	2	60.07	0	25.83
647	2	51.02	0	23.42
839	2	0.00	0.94	9.45
997	2	48.33	0	20.43
D3(BJ) Dispersion Corrected				
2	2	61.23	0	29.50
95	2	13.33	0	12.57
156	2	47.80	0	26.44
232	2	51.35	0	26.40
306	2	52.97	0	26.89
535	2	46.74	0	20.57
617	2	49.35	0	25.33
647	2	34.97	0	23.68
839	2	0.00	1	11.32
997	2	34.98	0	20.25

Table S5. ELR Candidate Structures CCS Performance for 6-31G(d)

Dispersion Uncorrected				
Conformation No.	Model	Relative Energy (kcal/mol)	Mol Fraction	CCS % Error
102	1	14.37	0	12.98
110	1	12.32	0	5.32
112	1	15.93	0	8.09
123	1	21.38	0	9.48
205	1	8.15	0	8.27
304	1	10.21	0	4.22
433	1	11.60	0	10.75
472	1	12.51	0	8.28
474	1	19.25	0	8.95
549	1	3.96	0	4.79
578	1	17.28	0	9.33
669	1	5.99	0	4.79
684	1	0.00	1	1.27
746	1	21.81	0	7.92
797	1	24.15	0	14.36
879	1	24.64	0	14.52
883	1	23.95	0	7.90
962	1	28.26	0	15.93
966	1	11.18	0	6.19
D3(0) Dispersion Corrected				

102	1	18.76	0	9.62
110	1	13.92	0	3.72
112	1	13.33	0	3.27
123	1	22.63	0	8.26
205	1	0.00	0.9	1.27
304	1	9.35	0	1.57
433	1	17.67	0	8.74
472	1	9.33	0	4.01
474	1	13.80	0	9.23
549	1	1.29	0.1	3.84
578	1	15.74	0	7.34
669	1	4.40	0	3.85
684	1	10.82	0	0.43
746	1	18.81	0	2.95
797	1	29.64	0	11.72
879	1	28.38	0	12.81
883	1	15.31	0	1.57
962	1	32.54	0	15.83
966	1	15.38	0	2.26
D3(BJ) Dispersion Corrected				
102	1	21.04	0	9.74
110	1	16.11	0	3.70
112	1	5.74	0	0.89
123	1	25.48	0	8.25
205	1	0.00	1	0.65
304	1	21.16	0	4.08
433	1	20.70	0	8.58
472	1	11.69	0	4.01
474	1	16.28	0	8.75
549	1	3.53	0	3.81
578	1	18.19	0	6.99
669	1	6.68	0	3.78
684	1	3.81	0	2.64
746	1	21.21	0	3.13
797	1	17.86	0	2.11
879	1	30.44	0	12.15
883	1	17.79	0	1.50
962	1	35.05	0	15.82
966	1	12.36	0	2.29

Table S6. EWTR Candidate Structures CCS Performance for 6-31G(d)

Dispersion Uncorrected				
Conformation No.	Model	Relative Energy (kcal/mol)	Mol Fraction	CCS % Error
14	5	29.92	0	12.78
34	5	8.55	0	13.90

90	5	30.56	0	17.58
112	5	31.71	0	10.60
123	5	25.57	0	11.66
126	5	22.08	0	15.63
197	5	48.50	0	14.97
315	5	38.89	0	19.22
472	5	0.00	0.98	8.03
474	5	2.21	0.02	14.50
669	5	28.28	0	11.00
684	5	49.79	0	20.53
703	5	12.84	0	13.33
727	5	85.32	0	25.80
771	5	15.22	0	9.42
807	5	35.68	0	19.69
879	5	41.82	0	8.33
950	5	33.92	0	13.60
D3(0) Dispersion Corrected				
14	5	37.80	0	10.58
34	5	14.54	0	9.19
90	5	21.76	0	11.86
112	5	37.98	0	9.67
123	5	30.92	0	9.90
126	5	43.05	0	12.38
197	5	26.16	0	6.77
315	5	39.14	0	14.63
472	5	0.00	1	5.06
474	5	21.24	0	9.36
669	5	79.55	0	17.76
684	5	77.04	0	15.82
703	5	28.57	0	8.50
727	5	96.66	0	19.50
771	5	26.25	0	9.43
807	5	57.03	0	22.81
879	5	39.81	0	7.94
950	5	38.21	0	8.98
D3(BJ) Dispersion Corrected				
14	5	36.13	0	10.93
34	5	12.97	0	5.64
90	5	21.00	0	12.03
112	5	32.99	0	10.60
123	5	0.00	0.58	6.20
126	5	37.96	0	12.90
197	5	42.53	0	10.23
315	5	49.85	0	14.46
472	5	0.19	0.42	4.83
474	5	13.85	0	9.82

669	5	19.01	0	8.81
684	5	50.57	0	19.80
703	5	18.82	0	8.63
727	5	62.34	0	14.17
771	5	25.07	0	9.24
807	5	50.77	0	16.59
879	5	41.27	0	7.94
950	5	47.35	0	11.01

Table S7. EYK Candidate Structures CCS Performance for 6-31G(d)

Conformation No.	Model	Relative Energy (kcal/mol)	Mol Fraction	CCS % Error
Dispersion Uncorrected				
14	1	35.12	0	19.33
34	1	22.22	0	5.11
102	1	32.87	0	10.01
122	1	23.22	0	8.13
123	1	27.44	0	7.42
137	1	17.98	0	5.13
234	1	17.90	0	6.50
273	1	12.02	0	6.82
302	1	19.38	0	3.51
304	1	27.49	0	14.01
306	1	40.58	0	16.73
315	1	16.20	0	6.14
472	1	26.03	0	9.14
474	1	15.77	0	8.08
535	1	26.14	0	8.29
549	1	38.37	0	18.10
684	1	10.55	0	6.03
797	1	15.72	0	9.29
879	1	29.94	0	12.30
883	1	0.00	1	4.96
961	1	12.69	0	6.63
D3(0) Dispersion Corrected				
14	1	16.79	0	4.53
34	1	7.33	0	2.72
102	1	36.55	0	9.91
122	1	23.47	0	4.78
123	1	30.23	0	6.62
137	1	34.13	0	10.09
234	1	1.61	0.06	3.81
273	1	19.36	0	6.45
302	1	20.37	0	3.06
304	1	33.51	0	12.64
306	1	43.14	0	12.32

315	1	14.98	0	2.65
472	1	34.00	0	11.93
474	1	15.45	0	1.31
535	1	26.05	0	3.12
549	1	44.80	0	17.82
684	1	11.58	0	4.99
797	1	18.03	0	8.46
879	1	34.17	0	10.11
883	1	0.00	0.94	3.52
961	1	14.17	0	4.92
D3(BJ) Dispersion Corrected				
14	1	13.02	0	3.86
34	1	6.88	0	2.65
102	1	19.39	0	4.73
122	1	37.70	0	9.47
123	1	29.92	0	6.28
137	1	16.54	0	3.27
234	1	10.05	0	5.75
273	1	37.43	0	8.82
302	1	19.74	0	2.88
304	1	33.19	0	12.43
306	1	43.01	0	12.29
315	1	10.54	0	0.45
472	1	33.73	0	12.02
474	1	16.34	0	5.23
535	1	14.56	0	1.14
549	1	44.62	0	17.89
684	1	10.76	0	4.72
797	1	17.36	0	8.34
879	1	34.42	0	12.07
883	1	0.00	1	3.14
961	1	13.35	0	4.58

Table S8. FAAYLER Candidate Structures CCS Performance for 6-31G(d)

Dispersion Uncorrected				
Conformation No.	Model	Relative Energy (kcal/mol)	Mol Fraction	CCS % Error
102	1	32.75	0	26.66
123	1	0.00	0.65	12.76
197	1	30.42	0	23.24
217	1	30.98	0	25.05
231	1	31.74	0	18.08
304	1	22.71	0	24.78
306	1	49.87	0	26.20
364	1	31.12	0	17.14
367	1	37.00	0	25.23

399	1	43.20	0	23.52
448	1	27.84	0	24.33
474	1	25.63	0	21.74
497	1	24.71	0	24.79
625	1	27.44	0	24.67
669	1	0.36	0.35	12.09
684	1	51.16	0	25.15
727	1	37.60	0	22.92
746	1	48.23	0	23.12
883	1	15.36	0	10.33
950	1	72.70	0	27.27
961	1	68.59	0	24.56
962	1	31.24	0	24.54
966	1	24.76	0	26.80
D3(0) Dispersion Corrected				
102	1	69.80	0	18.43
123	1	70.43	0	21.03
156	1	45.86	0	5.91
197	1	55.84	0	22.10
217	1	86.20	0	21.71
231	1	48.35	0	14.00
304	1	56.38	0	14.81
306	1	61.68	0	20.27
364	1	38.84	0	3.35
367	1	57.62	0	20.54
399	1	75.85	0	21.83
448	1	66.37	0	22.82
474	1	54.16	0	13.31
497	1	43.17	0	12.62
625	1	67.89	0	21.73
669	1	0.00	1	2.98
684	1	46.03	0	17.55
746	1	14.45	0	0.65
883	1	44.40	0	11.73
950	1	44.82	0	13.14
961	1	74.30	0	13.36
962	1	75.56	0	15.17
966	1	55.31	0	23.65
D3(BJ) Dispersion Corrected				
102	1	32.43	0	9.94
123	1	58.51	0	21.06
156	1	0.00	1	4.58
197	1	61.61	0	17.53
217	1	38.27	0	16.72
231	1	36.41	0	14.25
304	1	25.31	0	6.46

306	1	57.86	0	16.58
364	1	54.14	0	22.55
367	1	46.78	0	20.50
399	1	57.71	0	20.02
448	1	41.94	0	22.57
474	1	5.37	0	7.43
497	1	31.23	0	12.55
625	1	56.51	0	21.45
669	1	25.20	0	9.04
684	1	35.23	0	15.09
727	1	92.54	0	24.30
746	1	4.18	0	2.68
883	1	42.75	0	9.07
950	1	33.56	0	14.63
961	1	69.79	0	18.49
962	1	38.79	0	15.16
966	1	32.85	0	19.33

Table S9. FLNR Candidate Structures CCS Performance for 6-31G(d)

Dispersion Uncorrected				
Conformation No.	Model	Relative Energy (kcal/mol)	Mol Fraction	CCS % Error
34	2	10.34	0	12.02
95	2	21.32	0	18.37
102	2	26.76	0	16.96
123	2	7.86	0	15.42
197	2	26.07	0	19.10
242	2	1.99	0.03	7.60
535	2	4.81	0	10.24
549	2	15.28	0	11.00
605	2	2.19	0.02	9.41
669	2	9.43	0	12.58
684	2	24.46	0	15.70
727	2	14.75	0	16.26
746	2	20.74	0	10.73
777	2	18.84	0	8.05
797	2	30.92	0	19.21
879	2	6.08	0	9.18
883	2	0.00	0.95	7.35
962	2	3.72	0	7.82
D3(0) Dispersion Corrected				
34	2	15.68	0	10.73
95	2	31.28	0	16.89
102	2	41.19	0	17.09
123	2	44.85	0	19.03
197	2	35.63	0	14.13

242	2	16.85	0	3.60
535	2	22.30	0	10.18
549	2	9.80	0	7.20
605	2	9.77	0	3.99
669	2	14.07	0	9.56
684	2	30.83	0	11.63
727	2	22.65	0	14.57
746	2	25.80	0	9.64
777	2	0.00	1	0.22
797	2	40.14	0	16.45
879	2	11.28	0	6.43
883	2	8.37	0	2.05
962	2	3.08	0	2.88
D3(BJ) Dispersion Corrected				
34	2	13.25	0	10.69
95	2	28.53	0	16.57
102	2	34.89	0	16.19
123	2	42.77	0	19.21
197	2	2.14	0.03	12.94
242	2	14.23	0	3.68
535	2	16.32	0	7.49
549	2	14.71	0	10.21
605	2	2.99	0	1.00
669	2	33.49	0	15.13
684	2	28.12	0	11.65
727	2	19.85	0	14.73
746	2	23.22	0	9.34
777	2	18.30	0	2.99
797	2	37.35	0	16.27
879	2	6.62	0	7.09
883	2	5.59	0	2.16
962	2	0.00	0.97	2.61

Table S10. FPK Candidate Structures CCS Performance for 6-31G(d)

Dispersion Uncorrected				
Conformation No.	Model	Relative Energy (kcal/mol)	Mol Fraction	CCS % Error
883	2	0.55	0.28	3.09
876	2	0.00	0.72	4.98
222	2	15.66	0	6.26
635	2	5.77	0	7.16
684	2	36.10	0	7.47
445	2	25.73	0	8.22
746	2	27.94	0	8.69
578	2	31.66	0	8.74
62	2	24.27	0	9.30

962	2	6.42	0	9.43
304	2	9.51	0	9.49
474	2	11.66	0	12.00
123	2	9.34	0	12.15
34	2	8.22	0	12.48
102	2	19.04	0	13.56
950	2	16.42	0	13.92
472	2	18.55	0	14.14
497	2	30.25	0	14.32
981	2	30.61	0	14.79
797	2	15.01	0	14.95
535	2	29.15	0	15.14
879	2	38.00	0	19.10
D3(0) Dispersion Corrected				
34	2	14.47	0	11.10
62	2	27.57	0	7.60
102	2	23.80	0	11.00
123	2	18.75	0	8.49
222	2	17.58	0	2.50
304	2	16.86	0	7.58
445	2	28.36	0	6.29
472	2	29.07	0	7.79
474	2	21.08	0	10.99
497	2	5.80	0	4.53
535	2	34.75	0	14.24
578	2	34.15	0	7.21
635	2	8.14	0	6.73
684	2	34.65	0	6.17
746	2	25.60	0	4.46
797	2	23.32	0	13.54
876	2	30.79	0	6.02
879	2	35.73	0	14.53
883	2	0.00	1	2.03
950	2	30.60	0	13.91
962	2	10.65	0	8.31
981	2	35.88	0	11.62
D3(BJ) Dispersion Corrected				
34	2	14.71	0	11.17
62	2	27.93	0	7.40
102	2	23.61	0	10.95
123	2	19.57	0	8.42
222	2	46.11	0	15.63
304	2	13.95	0	8.27
445	2	28.43	0	6.75
472	2	29.17	0	7.27
474	2	8.27	0	8.15

497	2	5.98	0	4.65
535	2	34.86	0	14.41
578	2	34.54	0	7.20
635	2	8.16	0	6.55
684	2	34.63	0	5.93
746	2	25.57	0	4.24
797	2	23.46	0	13.56
876	2	29.67	0	5.12
879	2	35.83	0	14.24
883	2	0.00	1	2.06
950	2	24.48	0	12.96
962	2	9.12	0	7.75
981	2	35.28	0	11.01

Table S11. FSSDR Candidate Structures CCS Performance for 6-31G(d)

Dispersion Uncorrected				
Conformation No.	Model	Relative Energy (kcal/mol)	Mol Fraction	CCS % Error
90	1	13.66	0	12.99
112	1	11.87	0	16.78
121	1	6.97	0	17.67
156	1	21.54	0	20.54
217	1	7.00	0	12.87
234	1	12.53	0	12.75
304	1	13.00	0	14.31
399	1	0.00	1	6.17
420	1	8.24	0	17.17
472	1	17.59	0	16.20
474	1	17.52	0	16.04
549	1	34.77	0	26.15
641	1	39.51	0	22.99
669	1	19.75	0	19.49
684	1	15.40	0	17.26
797	1	3.19	0	16.51
876	1	17.62	0	13.08
879	1	32.35	0	21.51
883	1	26.64	0	13.94
962	1	14.56	0	9.92
D3(0) Dispersion Corrected				
90	1	22.77	0	10.03
112	1	13.50	0	13.92
121	1	10.64	0	16.90
156	1	29.20	0	20.26
217	1	28.67	0	14.94
234	1	17.46	0	12.06

304	1	12.85	0	12.60
399	1	13.73	0	7.49
420	1	36.72	0	21.90
472	1	26.95	0	13.22
474	1	19.76	0	12.00
549	1	44.61	0	24.98
641	1	44.22	0	21.17
669	1	25.50	0	17.67
684	1	16.11	0	11.93
797	1	4.63	0	7.37
876	1	0.00	1	6.52
879	1	22.66	0	16.90
883	1	26.53	0	12.55
962	1	11.63	0	5.00
D3(BJ) Dispersion Corrected				
90	1	25.55	0	11.05
112	1	24.18	0	14.08
121	1	21.24	0	16.61
156	1	39.90	0	19.86
217	1	40.52	0	14.48
234	1	28.19	0	12.18
304	1	23.99	0	12.77
399	1	0.00	1	2.39
420	1	26.06	0	14.72
472	1	27.54	0	11.57
474	1	21.18	0	9.99
549	1	55.88	0	24.62
641	1	55.48	0	21.33
669	1	36.42	0	17.75
684	1	27.81	0	12.21
797	1	16.23	0	7.15
876	1	13.56	0	9.63
879	1	48.99	0	19.47
883	1	38.39	0	12.47
962	1	16.33	0	6.24

Table S12. Poly-Glycine-6 Candidate Structures CCS Performance for 6-31G(d)

Dispersion Uncorrected				
Conformation No.	Model	Relative Energy (kcal/mol)	Mol Fraction	CCS % Error
34	1	10.07	0	13.54
35	1	0.97	0.11	3.31
95	1	24.38	0	22.48
112	1	25.26	0	17.42
197	1	25.26	0	26.29
315	1	21.98	0	25.20

428	1	14.92	0	18.29
472	1	18.37	0	19.05
538	1	27.13	0	18.38
549	1	29.35	0	24.76
584	1	5.55	0	7.46
607	1	28.14	0	25.34
669	1	32.40	0	15.69
684	1	0.00	0.57	3.17
705	1	31.97	0	23.74
727	1	34.96	0	25.80
797	1	14.11	0	8.33
824	1	6.39	0	9.19
879	1	0.35	0.32	4.18
883	1	19.36	0	12.97
961	1	3.22	0	4.28
D3(0) Dispersion Corrected				
34	1	4.71	0	7.02
35	1	50.59	0	21.17
95	1	39.73	0	21.98
112	1	14.40	0	5.05
197	1	43.77	0	25.98
315	1	40.83	0	25.27
428	1	15.99	0	13.09
472	1	31.34	0	18.80
538	1	3.86	0	0.84
549	1	46.09	0	24.53
584	1	5.64	0	3.07
607	1	45.44	0	25.09
669	1	41.80	0	15.45
684	1	3.21	0	0.91
705	1	19.75	0	12.08
727	1	42.83	0	17.33
797	1	18.83	0	7.38
824	1	0.00	1	1.87
879	1	8.68	0	4.35
883	1	26.13	0	9.81
961	1	6.38	0	3.77
D3(BJ) Dispersion Corrected				
34	1	6.37	0	6.71
35	1	14.92	0	0.48
95	1	40.52	0	22.01
112	1	16.29	0	5.03
197	1	44.25	0	25.88
315	1	41.24	0	25.06
428	1	17.18	0	12.70
472	1	32.44	0	18.44

538	1	6.05	0	0.57
549	1	46.89	0	24.50
584	1	8.63	0	3.72
607	1	46.11	0	25.02
669	1	43.51	0	14.10
684	1	0.00	1	0.59
705	1	21.00	0	12.01
727	1	43.76	0	17.19
797	1	20.67	0	7.28
824	1	8.96	0	1.48
879	1	10.77	0	4.53
883	1	27.92	0	9.79
961	1	8.64	0	3.69

Table S13. Poly-Glycine-8 Candidate Structures CCS Performance for 6-31G(d)

Dispersion Uncorrected				
Conformation No.	Model	Relative Energy (kcal/mol)	Mol Fraction	CCS % Error
34	1	25.85	0	19.12
60	1	18.88	0	21.31
64	1	39.66	0	30.47
84	1	24.82	0	27.18
90	1	32.87	0	31.33
102	1	30.94	0	19.72
123	1	35.38	0	25.46
137	1	8.91	0	9.36
156	1	33.95	0	29.77
197	1	36.28	0	27.80
222	1	17.59	0	15.92
231	1	37.19	0	25.79
302	1	36.61	0	31.87
304	1	14.08	0	10.60
306	1	3.36	0	11.08
315	1	27.61	0	21.74
394	1	27.13	0	23.76
472	1	11.72	0	10.52
474	1	35.56	0	24.33
510	1	12.91	0	12.47
521	1	18.85	0	18.67
549	1	6.70	0	9.02
578	1	19.56	0	22.32
607	1	10.56	0	8.61
617	1	42.60	0	26.85
684	1	15.57	0	16.70
771	1	0.00	1	4.51
961	1	8.66	0	16.23

962	1	16.48	0	15.85
D3(0) Dispersion Corrected				
34	1	39.37	0	17.80
60	1	30.54	0	16.09
64	1	64.07	0	30.24
84	1	47.41	0	26.95
90	1	59.47	0	31.21
102	1	23.88	0	7.32
123	1	54.71	0	23.53
137	1	16.03	0	8.32
156	1	56.18	0	28.71
197	1	58.99	0	27.28
222	1	61.48	0	26.30
231	1	55.53	0	23.54
302	1	0.00	0.93	3.63
304	1	16.87	0	6.67
306	1	21.53	0	12.53
315	1	71.45	0	27.36
394	1	46.27	0	23.12
472	1	41.36	0	13.16
474	1	26.61	0	7.40
510	1	21.58	0	11.69
521	1	34.00	0	16.23
549	1	21.83	0	8.24
578	1	33.71	0	18.88
607	1	6.45	0	4.20
617	1	45.53	0	20.72
684	1	34.29	0	13.89
771	1	1.52	0.07	2.76
961	1	17.93	0	15.18
962	1	10.42	0	4.34
D3(BJ) Dispersion Corrected				
34	1	37.77	0	17.78
60	1	32.15	0	20.63
64	1	61.62	0	30.23
84	1	45.02	0	26.92
90	1	51.65	0	29.32
102	1	24.65	0	9.74
123	1	52.88	0	23.66
137	1	14.90	0	8.29
156	1	5.23	0	4.49
197	1	57.11	0	27.25
222	1	29.89	0	13.98
231	1	56.82	0	23.02
302	1	42.53	0	21.29
304	1	15.65	0	6.80

306	1	13.95	0	7.90
315	1	37.67	0	19.39
394	1	44.25	0	23.16
472	1	30.05	0	8.69
474	1	25.46	0	7.59
510	1	20.23	0	11.49
521	1	32.07	0	16.11
549	1	20.65	0	7.16
578	1	37.09	0	19.87
607	1	16.84	0	6.51
617	1	17.94	0	1.92
684	1	33.50	0	13.76
771	1	0.00	1	1.74
961	1	21.34	0	15.26
962	1	10.06	0	4.66

Table S14. Poly-Glycine-10 Candidate Structures CCS Performance for 6-31G(d)

Dispersion Uncorrected				
Conformation No.	Model	Relative Energy (kcal/mol)	Mol Fraction	CCS % Error
34	1	18.14	0	18.36
81	1	24.26	0	18.28
90	1	9.21	0	7.01
211	1	22.66	0	20.39
217	1	17.89	0	15.94
234	1	41.01	0	22.66
242	1	16.34	0	7.86
351	1	1.33	0.09	4.78
446	1	16.98	0	8.05
474	1	53.51	0	25.00
497	1	23.97	0	11.51
510	1	3.32	0	11.13
538	1	19.46	0	14.43
549	1	49.77	0	31.83
574	1	14.95	0	13.09
684	1	8.86	0	11.40
796	1	32.54	0	27.63
876	1	15.67	0	12.16
879	1	31.56	0	21.61
883	1	29.27	0	14.99
961	1	44.41	0	33.68
962	1	0.00	0.91	7.17
D3(0) Dispersion Corrected				
34	1	32.81	0	16.39
81	1	30.50	0	16.52
90	1	13.90	0	4.19

211	1	0.00	1	2.73
217	1	9.19	0	4.40
234	1	5.12	0	1.70
242	1	29.70	0	13.20
351	1	8.11	0	6.79
446	1	9.22	0	4.35
474	1	15.72	0	4.36
497	1	11.09	0	5.43
510	1	20.39	0	9.41
538	1	21.00	0	6.54
549	1	17.90	0	6.55
574	1	45.47	0	13.29
684	1	26.68	0	18.08
796	1	52.91	0	27.26
876	1	42.66	0	11.57
879	1	13.29	0	9.16
883	1	37.70	0	13.85
961	1	72.19	0	32.99
962	1	23.34	0	7.61
D3(BJ) Dispersion Corrected				
34	1	81.84	0	28.86
81	1	30.88	0	16.19
90	1	14.08	0	3.87
211	1	0.00	1	2.48
217	1	25.97	0	6.00
234	1	5.51	0	1.56
242	1	22.22	0	8.90
351	1	28.56	0	10.90
446	1	8.90	0	4.19
474	1	38.92	0	12.90
497	1	10.84	0	5.76
510	1	20.21	0	9.34
538	1	20.98	0	6.78
549	1	17.75	0	6.44
574	1	19.10	0	8.09
684	1	45.67	0	18.79
796	1	50.76	0	27.14
876	1	9.76	0	2.25
879	1	18.89	0	10.24
883	1	36.79	0	13.79
961	1	34.38	0	21.53
962	1	15.53	0	6.12

Table S15. Poly-Glycine-14 Candidate Structures CCS Performance for 6-31G(d)

Conformation No.	Model	Relative Energy (kcal/mol)	Mol Fraction	CCS % Error
Dispersion Uncorrected				
12	1	4.94	0	14.45
145	1	24.69	0	26.83
156	1	0.00	0.65	7.36
172	1	20.18	0	16.16
202	1	42.15	0	23.51
205	1	2.78	0.01	10.29
242	1	0.37	0.34	10.24
333	1	27.57	0	22.87
497	1	11.74	0	13.35
558	1	27.34	0	16.67
578	1	36.67	0	21.32
584	1	40.62	0	25.57
702	1	30.52	0	15.54
746	1	18.18	0	14.82
D3(0) Dispersion Corrected				
12	1	0.00	0.99	4.49
145	1	37.81	0	22.47
156	1	2.71	0.01	2.16
172	1	14.14	0	6.79
202	1	20.22	0	10.32
205	1	50.33	0	16.44
242	1	15.31	0	4.52
333	1	51.49	0	17.51
497	1	30.33	0	10.05
558	1	10.51	0	9.29
578	1	24.88	0	11.33
584	1	50.74	0	18.66
702	1	36.73	0	10.06
746	1	30.73	0	7.09
D3(BJ) Dispersion Corrected				
12	1	25.52	0	8.78
145	1	40.71	0	22.34
156	1	6.76	0	2.33
172	1	17.53	0	6.85
202	1	47.18	0	16.15
205	1	57.68	0	16.27
242	1	31.82	0	6.41
333	1	44.40	0	15.33
497	1	25.52	0	7.66
558	1	0.00	1	4.44
578	1	48.27	0	12.06
584	1	50.84	0	18.29
702	1	42.82	0	12.27

746	1	34.78	0	5.91

Table S16. GLVK Candidate Structures CCS Performance for 6-31G(d)

Dispersion Uncorrected				
Conformation No.	Model	Relative Energy (kcal/mol)	Mol Fraction	CCS % Error
34	2	9.40	0	4.75
81	2	9.61	0	4.16
102	2	20.39	0	2.86
123	2	39.82	0	17.10
234	2	0.00	1	2.61
302	2	10.49	0	4.10
370	2	41.48	0	17.71
448	2	5.54	0	4.07
472	2	41.22	0	17.69
584	2	9.24	0	3.24
605	2	35.66	0	9.52
684	2	40.22	0	14.05
731	2	18.09	0	4.94
746	2	39.32	0	14.87
876	2	4.53	0	3.09
879	2	42.56	0	17.12
883	2	2.62	0.01	3.33
950	2	12.50	0	4.78
962	2	11.94	0	5.71
983	2	33.68	0	12.52
D3(0) Dispersion Corrected				
34	2	6.48	0	3.72
81	2	6.56	0	3.04
102	2	16.23	0	1.84
123	2	5.90	0	4.53
234	2	18.86	0	1.02
302	2	15.07	0	5.16
370	2	44.70	0	17.07
448	2	1.88	0.04	2.66
472	2	43.76	0	16.64
584	2	6.70	0	0.37
605	2	35.17	0	8.42
684	2	37.58	0	7.12
731	2	13.17	0	4.70
746	2	39.47	0	10.91
876	2	0.00	0.96	2.55
879	2	46.85	0	16.56
883	2	2.31	0	2.44
950	2	2.27	0	3.87
962	2	24.46	0	7.09

983	2	14.60	0	3.07
D3(BJ) Dispersion Corrected				
34	2	7.55	0	3.48
81	2	7.71	0	3.07
102	2	18.13	0	1.69
123	2	6.51	0	4.47
234	2	17.79	0	1.25
302	2	15.86	0	4.99
370	2	47.79	0	17.03
448	2	11.23	0	4.78
472	2	45.33	0	16.38
584	2	6.66	0	2.19
605	2	36.60	0	8.34
684	2	38.84	0	11.98
731	2	35.77	0	4.03
746	2	41.16	0	11.09
876	2	1.60	0.06	2.45
879	2	6.64	0	4.52
883	2	0.00	0.94	2.53
950	2	3.21	0	3.93
962	2	9.90	0	4.56
983	2	15.43	0	2.87

Table S17. LWSAK Candidate Structures CCS Performance for 6-31G(d)

Dispersion Uncorrected				
Conformation No.	Model	Relative Energy (kcal/mol)	Mol Fraction	CCS % Error
18	1	53.05	0	24.80
34	1	54.95	0	23.44
102	1	3.58	0	18.91
112	1	20.18	0	9.03
231	1	5.47	0	20.26
288	1	2.45	0.01	17.71
315	1	40.13	0	20.10
428	1	48.65	0	20.48
459	1	12.75	0	14.15
472	1	5.03	0	20.77
474	1	7.05	0	14.02
549	1	14.01	0	20.11
669	1	0.61	0.26	17.73
684	1	0.00	0.73	8.56
727	1	9.23	0	19.68
746	1	39.88	0	17.43
771	1	12.95	0	16.79
772	1	10.37	0	21.31
879	1	4.25	0	19.53

950	1	4.19	0	17.03
961	1	39.08	0	9.79
962	1	6.93	0	17.80
D3(0) Dispersion Corrected				
18	1	81.12	0	23.73
34	1	80.21	0	19.72
102	1	25.67	0	10.33
112	1	50.40	0	7.66
231	1	38.50	0	14.19
288	1	59.00	0	15.38
315	1	63.61	0	19.89
428	1	62.21	0	15.92
459	1	18.01	0	7.26
472	1	42.67	0	15.28
474	1	30.79	0	10.56
549	1	50.16	0	17.82
669	1	25.82	0	12.15
684	1	0.00	1	2.34
727	1	37.00	0	18.75
746	1	62.46	0	14.18
771	1	37.83	0	15.53
772	1	16.28	0	9.73
879	1	37.02	0	16.63
950	1	68.14	0	15.92
961	1	45.63	0	2.72
962	1	36.10	0	15.89
D3(BJ) Dispersion Corrected				
18	1	82.54	0	23.71
34	1	82.60	0	19.83
102	1	27.62	0	10.15
112	1	52.10	0	7.39
231	1	34.66	0	14.75
288	1	21.00	0	10.98
315	1	65.24	0	19.53
428	1	73.19	0	18.34
459	1	20.65	0	7.15
472	1	96.03	0	24.11
474	1	33.10	0	10.69
549	1	47.48	0	17.45
669	1	28.13	0	12.17
684	1	0.00	1	2.43
727	1	38.89	0	18.84
746	1	66.64	0	13.12
771	1	40.36	0	15.37
772	1	19.44	0	9.66
879	1	36.00	0	17.43

950	1	31.41	0	9.13
961	1	74.95	0	10.36
962	1	37.61	0	15.77

Table S18. NFNR Candidate Structures CCS Performance for 6-31G(d)

Conformation No.	Model	Relative Energy (kcal/mol)	Mol Fraction	CCS % Error
Dispersion Uncorrected				
34	2	5.46	0	13.43
102	2	14.45	0	11.75
112	2	19.72	0	18.04
123	2	25.24	0	15.04
156	2	10.52	0	15.00
433	2	7.79	0	9.90
472	2	32.27	0	13.35
549	2	16.80	0	15.39
684	2	13.24	0	13.37
727	2	23.67	0	20.42
746	2	5.85	0	6.77
772	2	12.14	0	12.32
879	2	0.00	0.51	11.99
883	2	15.35	0	14.72
950	2	23.51	0	19.00
962	2	0.01	0.49	10.56
D3(0) Dispersion Corrected				
34	2	8.06	0	7.41
102	2	10.32	0	9.16
112	2	20.09	0	16.58
123	2	24.66	0	13.47
156	2	17.94	0	12.98
433	2	2.65	0.01	10.39
472	2	32.56	0	14.30
549	2	16.01	0	11.44
684	2	11.89	0	9.13
727	2	22.08	0	13.16
746	2	22.86	0	9.63
772	2	18.94	0	10.29
879	2	3.56	0	5.60
883	2	9.20	0	10.83
950	2	19.31	0	14.67
962	2	0.00	0.99	9.53
D3(BJ) Dispersion Corrected				
34	2	0.00	0.99	6.60
102	2	17.33	0	9.31
123	2	30.69	0	13.52
156	2	23.90	0	12.50

433	2	10.12	0	7.75
472	2	8.10	0	1.25
549	2	2.49	0.01	9.18
684	2	17.89	0	8.79
727	2	19.11	0	10.62
746	2	11.18	0	4.78
772	2	14.23	0	6.55
879	2	7.31	0	5.08
883	2	25.78	0	9.91
950	2	13.23	0	9.12
962	2	23.99	0	11.85

Table S19. NIATSGK Candidate Structures CCS Performance for 6-31G(d)

Dispersion Uncorrected				
Conformation No.	Model	Relative Energy (kcal/mol)	Mol Fraction	CCS % Error
14	4	42.39	0	23.72
34	4	59.90	0	15.61
102	4	33.29	0	15.99
112	4	16.98	0	16.37
123	4	33.13	0	23.50
137	4	10.33	0	12.72
156	4	30.46	0	19.41
205	4	9.26	0	14.23
234	4	14.05	0	16.08
302	4	29.22	0	11.05
448	4	5.44	0	8.78
472	4	18.37	0	14.12
496	4	28.87	0	22.86
535	4	23.10	0	16.62
553	4	0.00	1	14.54
746	4	20.90	0	17.66
841	4	11.58	0	7.94
879	4	3.40	0	15.69
883	4	41.80	0	17.30
950	4	30.47	0	17.62
962	4	32.95	0	22.34
D3(0) Dispersion Corrected				
112	4	68.42	0	21.31
121	4	45.44	0	22.06
197	4	53.42	0	18.83
234	4	37.12	0	6.52
420	4	41.14	0	11.75
472	4	25.58	0	8.96
538	4	42.08	0	8.21
669	4	80.40	0	15.06

727	4	44.05	0	11.65
771	4	43.80	0	7.54
772	4	21.89	0	5.44
797	4	58.83	0	15.40
807	4	86.99	0	18.47
828	4	0.00	1	1.03
879	4	54.40	0	8.04
883	4	72.80	0	24.84
950	4	45.58	0	18.30
962	4	68.02	0	20.78
D3(BJ) Dispersion Corrected				
112	4	64.87	0	21.32
121	4	40.73	0	21.71
197	4	50.01	0	18.57
234	4	16.91	0	6.52
420	4	27.03	0	9.92
472	4	26.86	0	10.67
538	4	39.45	0	8.04
669	4	32.61	0	0.22
727	4	33.37	0	8.20
771	4	76.84	0	19.26
772	4	19.30	0	5.33
797	4	55.76	0	15.76
807	4	51.71	0	13.49
828	4	0.00	1	2.66
879	4	51.54	0	8.54
883	4	33.95	0	16.59
950	4	42.55	0	18.44
962	4	57.82	0	17.24

Table S20. TFAEALR Candidate Structures CCS Performance for 6-31G(d)

Dispersion Uncorrected				
Conformation No.	Model	Relative Energy (kcal/mol)	Mol Fraction	CCS % Error
772	3	0	0.98	13.88
242	3	2.43	0.02	14.05
684	3	4.67	0	15.28
448	3	4.92	0	17.13
535	3	10.14	0	19.84
329	3	11.74	0	16.58
474	3	13.24	0	18.71
445	3	13.3	0	17.12
156	3	13.62	0	17.54
304	3	14.73	0	16.38
771	3	15.67	0	19.5

727	3	15.94	0	20.1
883	3	18.68	0	20.37
746	3	20.18	0	20.58
112	3	20.66	0	12.99
879	3	21.27	0	23.54
472	3	23.24	0	18.98
549	3	24.92	0	20.39
D3(0) Dispersion Corrected				
883	3	0	1	5.11
549	3	17.98	0	8.26
771	3	19.28	0	15.89
472	3	20.12	0	13.04
242	3	20.86	0	5.12
445	3	26.19	0	14.7
746	3	26.68	0	3.09
329	3	27.7	0	14.78
304	3	28.14	0	14.43
448	3	28.67	0	16.11
112	3	29.72	0	17.72
474	3	30.48	0	9.08
727	3	31.97	0	16.97
535	3	33.02	0	13.41
156	3	33.83	0	8.09
684	3	39.84	0	17.52
772	3	44.48	0	16.51
879	3	46.72	0	22.41
D3(BJ) Dispersion Corrected				
772	3	0	1	3.57
746	3	11.55	0	1
883	3	13.24	0	6.95
242	3	21.95	0	6.73
252	3	23.18	0	15.53
472	3	24.48	0	13.1
112	3	25.31	0	11.23
329	3	29	0	15.89
445	3	30.27	0	14.55
304	3	32.18	0	14.08
448	3	32.66	0	15.9
771	3	34.64	0	15.51
727	3	36.12	0	16.85
535	3	38.35	0	14.39
474	3	38.84	0	12
684	3	43.73	0	17.18
156	3	44.94	0	13.6
549	3	45.64	0	13.4

Table S21. TIAQYAR Candidate Structures CCS Performance for 6-31G(d)

Conformation No.	Model	Relative Energy (kcal/mol)	Mol Fraction	CCS % Error
Dispersion Uncorrected				
34	1	7.94	0	22.25
123	1	15.04	0	26.12
156	1	12.81	0	23.03
234	1	15.05	0	26.07
242	1	28.84	0	18.93
364	1	14.09	0	15.71
448	1	46.24	0	23.85
450	1	16.42	0	24.57
472	1	0	1	19.12
474	1	6.29	0	21.27
535	1	55.11	0	27.23
549	1	16.95	0	28.57
578	1	21.7	0	24.15
584	1	50.98	0	28.79
662	1	39.03	0	24.3
746	1	9.94	0	25.81
772	1	12.84	0	22.88
876	1	43.12	0	25.69
883	1	25.91	0	22.21
961	1	10.23	0	19.59
962	1	28.32	0	21.32
966	1	7.3	0	18.74
D3(0) Dispersion Corrected				
34	1	31.38	0	15.87
123	1	43.41	0	25.22
156	1	37.10	0	21.54
234	1	65.49	0	23.54
242	1	66.66	0	21.72
364	1	18.85	0	11.45
448	1	14.51	0	8.68
450	1	42.80	0	22.05
472	1	39.19	0	18.34
474	1	46.38	0	18.57
535	1	81.94	0	25.32
549	1	34.22	0	21.01
578	1	7.60	0	1.13
584	1	81.94	0	27.07
662	1	52.41	0	21.54
746	1	35.73	0	18.33
772	1	33.93	0	19.92
876	1	62.56	0	23.89
883	1	65.08	0	19.71
961	1	28.34	0	18.97

962	1	0.00	1	0.74
966	1	18.10	0	14.61
D3(BJ) Dispersion Corrected				
34	1	15.51	0	13.81
123	1	36.23	0	25.19
156	1	30.57	0	21.56
234	1	24.02	0	17.54
242	1	60.51	0	21.51
364	1	7.19	0	10.74
448	1	10.88	0	9.14
450	1	36.13	0	22.05
472	1	24.79	0	19.51
474	1	42.86	0	19.16
535	1	74.32	0	25.01
549	1	18.15	0	16.74
584	1	75.79	0	27.11
662	1	53.13	0	19.91
746	1	6.87	0	9.32
772	1	24.42	0	16.29
876	1	56.42	0	23.75
883	1	0.00	0.99	6.89
961	1	2.94	0.01	15.27
962	1	12.89	0	5.33
966	1	11.52	0	15.70

Table S22. VASLR Candidate Structures CCS Performance for 6-31G(d)

Dispersion Uncorrected				
Conformation No.	Model	Relative Energy (kcal/mol)	Mol Fraction	CCS % Error
914	1	0.00	1	0.59
242	1	18.98	0	9.82
126	1	20.35	0	12.77
315	1	19.99	0	14.55
796	1	40.91	0	15.44
883	1	22.39	0	15.52
586	1	40.25	0	15.76
961	1	52.60	0	15.76
771	1	24.37	0	15.90
472	1	26.98	0	15.93
34	1	32.93	0	16.31
231	1	35.91	0	16.66
112	1	32.23	0	16.99
684	1	29.75	0	17.40
879	1	28.28	0	17.47
123	1	57.70	0	17.64
746	1	49.74	0	18.06

474	1	27.52	0	18.76
549	1	23.46	0	19.45
966	1	35.39	0	20.43
D3(0) Dispersion Corrected				
34	1	40.12	0	14.70
112	1	30.02	0	9.90
123	1	61.67	0	7.49
126	1	34.85	0	17.85
231	1	73.31	0	13.96
242	1	67.11	0	12.84
315	1	34.33	0	11.65
472	1	33.52	0	14.84
474	1	36.18	0	18.59
549	1	38.14	0	18.99
586	1	40.81	0	13.62
684	1	43.46	0	17.03
746	1	56.73	0	17.45
771	1	41.31	0	10.81
796	1	29.22	0	10.22
879	1	34.85	0	13.32
883	1	41.70	0	10.14
914	1	14.03	0	2.18
961	1	0.00	1	3.85
966	1	45.59	0	19.85
D3(BJ) Dispersion Corrected				
34	1	38.08	0	12.79
112	1	19.82	0	9.54
123	1	52.29	0	7.26
126	1	14.73	0	7.81
231	1	51.80	0	14.58
242	1	43.94	0	14.92
315	1	24.87	0	11.41
472	1	23.58	0	14.78
474	1	25.97	0	18.37
549	1	27.92	0	18.87
586	1	31.85	0	13.52
684	1	27.35	0	16.25
746	1	46.90	0	17.11
771	1	0.00	1	0.02
796	1	28.35	0	8.71
879	1	24.73	0	13.40
883	1	16.79	0	14.60
914	1	4.99	0	2.49
961	1	47.08	0	15.17
966	1	35.09	0	19.78

Table S23. WIR Candidate Structures CCS Performance for 6-31G(d)

Conformation No.	Model	Relative Energy (kcal/mol)	Mol Fraction	CCS % Error
Dispersion Uncorrected				
34	5	67.99	0	14.02
112	5	72.25	0	14.06
123	5	51.16	0	7.07
156	5	46.33	0	10.34
217	5	14.27	0	7.60
234	5	0.00	1	2.93
304	5	70.22	0	12.20
472	5	36.01	0	5.11
474	5	9.25	0	6.87
578	5	15.28	0	4.61
600	5	22.95	0	6.24
605	5	9.72	0	5.28
617	5	36.33	0	6.39
684	5	45.81	0	10.28
727	5	52.35	0	11.74
746	5	17.86	0	3.95
879	5	52.87	0	6.62
883	5	55.83	0	10.25
950	5	51.80	0	7.18
962	5	16.07	0	6.47
D3(0) Dispersion Corrected				
34	5	67.82	0	13.63
112	5	73.36	0	13.64
123	5	50.19	0	6.46
156	5	45.97	0	9.67
217	5	9.28	0	5.92
234	5	12.93	0	3.12
304	5	66.85	0	10.19
472	5	53.56	0	11.76
474	5	0.00	1	2.01
578	5	62.64	0	4.01
600	5	20.51	0	4.77
605	5	4.04	0	3.05
617	5	28.79	0	6.03
684	5	86.08	0	16.33
727	5	52.31	0	7.72
746	5	17.71	0	0.71
879	5	33.91	0	3.81
883	5	54.33	0	9.86
950	5	42.85	0	5.51
962	5	13.52	0	3.89
D3(BJ) Dispersion Corrected				
34	5	75.68	0	11.70

112	5	85.50	0	13.42
123	5	54.40	0	5.42
156	5	54.89	0	9.61
217	5	18.95	0	5.83
234	5	0.00	1	2.28
304	5	75.10	0	10.16
472	5	62.66	0	8.80
474	5	9.20	0	1.35
578	5	71.07	0	3.58
600	5	30.10	0	4.61
605	5	13.76	0	3.13
617	5	37.52	0	5.78
684	5	80.04	0	13.96
727	5	61.67	0	7.62
746	5	27.09	0	0.68
879	5	42.28	0	3.70
883	5	63.32	0	9.62
950	5	51.90	0	5.38
962	5	25.77	0	3.71

Figure S1. The final predicted gas phase charge state/protomer for peptide ions in positive mode, [M+H]+ . In this single-letter amino acid code form, the amino acid containing the positive charge, negative charge, or both is colored in blue, red, or purple, respectively.

Amino Acid Residue Charge Site

Positive Negative Both

1	**ANELLINVK**	9	**FLNR**	17	**LWSAK**
2	**AWEVTVK**	10	**FPK**	18	**NIATSGK**
3	**AWSVAR**	11	**FSSDR**	19	**TFAEALR**
4	**DYYFALAHTVR**	12	**GGGGGG**	20	**TIAQYAR**
5	**ELR**	13	**GGGGGGGG**	21	**VASLR**
6	**EWTR**	14	**GGGGGGGGGG**	22	**VSALYK**
7	**EYK**	15	**GGGGGGGGGGGGGG**	23	**WIR**
8	**FAAYLER**	16	**GLVK**		

Table S24. ANELLINVK CCS Performance with *p*-type function

D3(0)-B3LYP/6-31G(d,p)				
Conformation No.	Model	Relative Energy (kcal/mol)	Mol Fraction	CCS % Error
112	1	90.53	0	24.46
234	1	6.19	0	2.13
315	1	145.26	0	28.95
472	1	138.56	0	29.13
474	1	67.36	0	24.53
535	1	57.90	0	22.70
549	1	0.00	1	3.99
584	1	7.18	0	4.79
607	1	65.35	0	22.32
684	1	96.94	0	26.86
727	1	69.09	0	24.16
962	1	39.97	0	11.52
966	1	54.35	0	21.22
D3(BJ)-B3LYP/6-31G(d,p)				
Conformation No.	Model	Relative Energy (kcal/mol)	Mol Fraction	CCS % Error
112	1	74.61	0	22.40
234	1	77.74	0	23.30
315	1	53.36	0	20.42
472	1	130.88	0	29.15
474	1	62.21	0	24.39
535	1	29.92	0	12.90
549	1	30.98	0	10.92
584	1	0.00	1	2.92
607	1	60.18	0	22.27
684	1	90.32	0	26.32
727	1	58.98	0	22.77
962	1	50.99	0	16.27
966	1	54.60	0	25.06

Table S25. AWEVTVK CCS Performance with *p*-type function

D3(0)-B3LYP/6-31G(d,p)				
Conformation No.	Model	Relative Energy (kcal/mol)	Mol Fraction	CCS % Error
34	2	7.55	0	5.62
231	2	29.34	0	17.69
234	2	150.44	0	30.23
329	2	145.18	0	27.02
472	2	24.09	0	13.90
497	2	153.83	0	29.66
684	2	0.00	0.82	2.07
727	2	158.64	0	24.82
731	2	64.40	0	15.83

772	2	0.90	0.18	5.65
876	2	162.25	0	30.01
879	2	36.67	0	17.09
883	2	4.50	0	11.41
962	2	173.46	0	27.51
D3(BJ)-B3LYP/6-31G(d,p)				
Conformation No.	Model	Relative Energy (kcal/mol)	Mol Fraction	CCS % Error
34	2	12.10	0	12.58
231	2	26.33	0	17.18
234	2	148.27	0	30.02
329	2	33.98	0	15.12
472	2	15.03	0	12.49
497	2	152.20	0	29.66
684	2	64.50	0	19.74
727	2	32.92	0	13.54
731	2	62.07	0	15.77
772	2	0.00	1	5.51
876	2	160.22	0	30.01
879	2	31.78	0	15.05
883	2	126.78	0	28.26
962	2	170.95	0	27.51

Table S26. AWSVAR CCS Performance with *p*-type function

D3(0)-B3LYP/6-31G(d,p)				
Conformation No.	Model	Relative Energy (kcal/mol)	Mol Fraction	CCS % Error
34	2	44.82	0	17.38
102	2	28.62	0	9.47
121	2	9.76	0	10.44
126	2	17.43	0	10.31
197	2	12.66	0	11.52
217	2	19.58	0	15.77
239	2	7.88	0	10.59
418	2	23.65	0	19.34
448	2	16.40	0	14.14
472	2	11.50	0	14.38
474	2	18.27	0	9.33
496	2	39.58	0	18.67
549	2	30.88	0	21.37
662	2	25.58	0	17.20
669	2	15.90	0	14.92
684	2	26.30	0	13.22
727	2	0.00	1	6.87
879	2	39.40	0	21.24
961	2	10.49	0	10.04
962	2	14.11	0	2.15

966	2	7.56	0	10.55
		D3(BJ)-B3LYP/6-31G(d,p)		
Conformation No.	Model	Relative Energy (kcal/mol)	Mol Fraction	CCS % Error
34	2	52.69	0	17.10
102	2	35.18	0	6.29
121	2	18.05	0	10.31
126	2	25.16	0	10.21
197	2	20.18	0	11.73
217	2	27.53	0	15.51
239	2	21.13	0	14.07
472	2	19.40	0	14.29
474	2	26.45	0	8.88
496	2	22.40	0	10.72
549	2	38.38	0	21.19
662	2	33.23	0	16.60
669	2	23.51	0	14.89
684	2	34.12	0	13.19
727	2	8.58	0	6.65
879	2	46.89	0	21.49
961	2	14.59	0	6.64
962	2	10.10	0	4.46
966	2	0.00	1	3.55

Table S27. DYYFALAHTVR CCS Performance with *p*-type function

		D3(0)-B3LYP/6-31G(d,p)		
Conformation No.	Model	Relative Energy (kcal/mol)	Mol Fraction	CCS % Error
2	2	34.68	0	24.29
95	2	0.00	0.68	1.03
156	2	61.91	0	26.76
232	2	103.25	0	30.30
306	2	54.59	0	24.47
535	2	58.62	0	22.77
617	2	61.56	0	27.89
647	2	71.06	0	22.72
839	2	0.45	0.32	9.18
997	2	83.56	0	25.72
		D3(BJ)-B3LYP/6-31G(d,p)		
Conformation No.	Model	Relative Energy (kcal/mol)	Mol Fraction	CCS % Error
2	2	44.43	0	23.58
95	2	38.86	0	13.88
156	2	0.00	1	4.42
232	2	135.81	0	31.16
306	2	67.98	0	25.36
535	2	64.40	0	20.66

617	2	64.74	0	25.01
647	2	75.42	0	21.66
839	2	16.51	0	9.47
997	2	54.18	0	20.03

Table S28. ELR CCS Performance with *p*-type function

D3(0)-B3LYP/6-31G(d,p)				
Conformation No.	Model	Relative Energy (kcal/mol)	Mol Fraction	CCS % Error
102	1	17.37	0.00	9.64
110	1	12.41	0.00	3.56
112	1	12.04	0.00	2.75
123	1	21.49	0.00	7.99
205	1	12.61	0.00	5.03
304	1	6.50	0.00	2.57
433	1	16.61	0.00	8.04
472	1	8.00	0.00	3.86
474	1	12.35	0.00	8.65
549	1	0.00	1.00	3.67
578	1	14.53	0.00	7.06
669	1	3.14	0.00	3.75
684	1	6.41	0.00	0.45
746	1	17.62	0.00	2.58
797	1	28.49	0.00	11.66
879	1	27.26	0.00	12.57
883	1	14.15	0.00	1.30
962	1	31.20	0.00	15.30
966	1	14.84	0.00	5.66
D3(BJ)-B3LYP/6-31G(d,p)				
Conformation No.	Model	Relative Energy (kcal/mol)	Mol Fraction	CCS % Error
102	1	17.35	0.00	9.91
110	1	12.36	0.00	3.33
112	1	1.41	0.08	1.63
123	1	22.11	0.00	7.83
205	1	12.81	0.00	5.20
433	1	13.87	0.00	8.35
472	1	8.13	0.00	3.71
474	1	12.62	0.00	8.29
549	1	0.00	0.91	3.46
578	1	14.76	0.00	6.86
669	1	3.19	0.00	3.52
684	1	11.01	0.00	1.40
746	1	17.77	0.00	2.57
797	1	14.21	0.00	1.93
879	1	27.42	0.00	12.45
883	1	14.43	0.00	1.13

962	1	31.49	0.00	15.54
966	1	14.28	0.00	2.03

Table S29. EWTR CCS Performance with *p*-type function

D3(0)-B3LYP/6-31G(d,p)				
Conformation No.	Model	Relative Energy (kcal/mol)	Mol Fraction	CCS % Error
14	5	45.30	0.00	12.19
34	5	36.48	0.00	7.57
90	5	33.28	0.00	9.72
112	5	38.69	0.00	8.44
123	5	7.33	0.00	5.50
126	5	28.35	0.00	6.05
197	5	31.05	0.00	10.74
315	5	45.43	0.00	12.30
472	5	9.57	0.00	4.56
474	5	0.00	1.00	3.26
669	5	26.38	0.00	6.85
684	5	43.25	0.00	16.78
703	5	32.82	0.00	7.12
727	5	106.59	0.00	19.38
771	5	27.58	0.00	6.22
807	5	56.62	0.00	16.09
879	5	45.67	0.00	6.20
950	5	47.42	0.00	8.73
D3(BJ)-B3LYP/6-31G(d,p)				
Conformation No.	Model	Relative Energy (kcal/mol)	Mol Fraction	CCS % Error
14	5	29.01	0.00	7.35
34	5	41.61	0.00	3.13
90	5	32.91	0.00	11.47
112	5	48.67	0.00	10.02
126	5	28.02	0.00	6.04
197	5	16.32	0.00	5.86
315	5	45.13	0.00	12.13
472	5	10.51	0.00	2.50
474	5	0.00	1.00	3.09
669	5	26.39	0.00	7.61
684	5	58.34	0.00	19.45
703	5	32.45	0.00	6.68
727	5	54.30	0.00	4.96
771	5	26.85	0.00	5.73
807	5	64.11	0.00	22.61
879	5	36.05	0.00	4.05
950	5	33.58	0.00	8.47

Table S30. EYK CCS Performance with *p*-type function

Conformation No.	Model	Relative Energy (kcal/mol)	Mol Fraction	CCS % Error
D3(0)-B3LYP/6-31G(d,p)				
14	1	36.96	0.00	13.55
34	1	17.56	0.00	2.31
102	1	36.59	0.00	9.49
122	1	23.73	0.00	5.08
123	1	30.52	0.00	6.25
137	1	34.19	0.00	9.67
234	1	4.19	0.00	4.06
273	1	15.42	0.00	5.65
302	1	20.51	0.00	2.67
304	1	33.30	0.00	12.23
306	1	43.60	0.00	11.68
315	1	7.10	0.00	0.83
472	1	33.37	0.00	11.22
474	1	16.48	0.00	6.59
535	1	26.12	0.00	2.84
549	1	45.27	0.00	17.67
684	1	11.38	0.00	4.75
797	1	17.74	0.00	8.00
879	1	34.37	0.00	9.74
883	1	0.00	1.00	3.12
961	1	13.80	0.00	4.37
D3(BJ)-B3LYP/6-31G(d,p)				
Conformation No.	Model	Relative Energy (kcal/mol)	Mol Fraction	CCS % Error
14	1	12.76	0.00	3.67
34	1	6.75	0.00	2.14
102	1	16.66	0.00	2.90
122	1	22.95	0.00	4.61
123	1	30.20	0.00	6.04
137	1	16.66	0.00	2.69
234	1	4.49	0.00	3.85
273	1	57.16	0.00	15.13
302	1	19.87	0.00	2.27
304	1	32.95	0.00	12.06
306	1	43.49	0.00	11.64
315	1	14.64	0.00	2.11
472	1	29.94	0.00	7.69
474	1	15.81	0.00	6.21
535	1	14.31	0.00	0.94
549	1	45.09	0.00	17.52
684	1	10.53	0.00	4.44
797	1	17.05	0.00	7.85
879	1	34.47	0.00	11.73
883	1	0.00	1.00	2.78

961	1	12.92	0.00	4.02

Table S31. FAAYLER CCS Performance with *p*-type function

D3(0)-B3LYP/6-31G(d,p)				
Conformation No.	Model	Relative Energy (kcal/mol)	Mol Fraction	CCS % Error
102	1	74.74	0.00	20.47
123	1	74.44	0.00	20.92
156	1	37.79	0.00	9.17
197	1	66.77	0.00	14.54
217	1	42.85	0.00	7.47
231	1	55.31	0.00	14.58
304	1	36.07	0.00	9.88
306	1	64.64	0.00	18.10
364	1	48.61	0.00	7.78
367	1	61.19	0.00	20.01
399	1	85.19	0.00	19.30
448	1	70.86	0.00	22.51
474	1	19.60	0.00	7.58
497	1	46.38	0.00	12.14
625	1	71.27	0.00	22.60
669	1	0.00	1.00	0.11
684	1	50.99	0.00	17.11
727	1	107.57	0.00	24.15
746	1	21.39	0.00	1.14
883	1	42.19	0.00	9.75
961	1	92.58	0.00	19.73
962	1	74.67	0.00	13.71
966	1	60.10	0.00	23.38
D3(BJ)-B3LYP/6-31G(d,p)				
Conformation No.	Model	Relative Energy (kcal/mol)	Mol Fraction	CCS % Error
102	1	44.52	0.00	13.63
123	1	59.18	0.00	20.66
156	1	34.97	0.00	6.94
197	1	64.40	0.00	21.19
217	1	50.14	0.00	19.54
231	1	41.82	0.00	14.61
304	1	29.28	0.00	10.41
306	1	58.96	0.00	16.45
364	1	45.64	0.00	11.68
367	1	47.03	0.00	20.12
399	1	69.35	0.00	20.60
448	1	52.65	0.00	22.67
474	1	5.25	0.00	9.34
497	1	61.81	0.00	13.93
625	1	49.83	0.00	21.58

669	1	2.10	0.03	7.74
684	1	40.86	0.00	17.27
727	1	93.73	0.00	24.03
746	1	0.00	0.97	2.25
883	1	28.49	0.00	9.16
961	1	70.88	0.00	18.13
962	1	44.31	0.00	16.69
966	1	34.47	0.00	19.30

Table S32. FLNR CCS Performance with *p*-type function

D3(0)-B3LYP/6-31G(d,p)				
Conformation No.	Model	Relative Energy (kcal/mol)	Mol Fraction	CCS % Error
34	2	13.26	0.00	10.97
95	2	28.29	0.00	16.83
102	2	34.09	0.00	16.13
123	2	41.27	0.00	18.97
197	2	32.76	0.00	13.92
242	2	14.10	0.00	3.43
535	2	19.50	0.00	9.73
549	2	14.22	0.00	9.95
605	2	2.12	0.02	6.57
669	2	11.06	0.00	8.89
684	2	27.95	0.00	11.40
727	2	19.64	0.00	14.38
746	2	22.87	0.00	9.11
777	2	1.41	0.08	0.82
797	2	37.33	0.00	16.22
879	2	12.78	0.00	5.62
883	2	5.32	0.00	1.70
962	2	0.00	0.89	2.70
D3(BJ)-B3LYP/6-31G(d,p)				
Conformation No.	Model	Relative Energy (kcal/mol)	Mol Fraction	CCS % Error
34	2	13.85	0.00	10.97
95	2	28.62	0.00	16.36
102	2	39.30	0.00	16.64
123	2	40.07	0.00	19.49
197	2	2.65	0.01	11.70
242	2	14.57	0.00	3.50
535	2	28.55	0.00	10.95
549	2	7.57	0.00	6.75
605	2	2.46	0.02	6.52
669	2	33.80	0.00	14.88
684	2	28.24	0.00	11.11
727	2	19.90	0.00	14.55
746	2	23.38	0.00	8.96

777	2	18.74	0.00	2.60
797	2	37.60	0.00	15.99
879	2	13.68	0.00	6.37
883	2	5.63	0.00	1.63
962	2	0.00	0.97	2.34

Table S33. FPK CCS Performance with *p*-type function

D3(0)-B3LYP/6-31G(d,p)				
Conformation No.	Model	Relative Energy (kcal/mol)	Mol Fraction	CCS % Error
34	2	13.99	0.00	10.74
62	2	27.68	0.00	7.34
102	2	23.46	0.00	10.65
123	2	18.47	0.00	8.35
222	2	17.29	0.00	2.35
304	2	16.89	0.00	7.21
445	2	28.50	0.00	6.15
472	2	29.12	0.00	7.57
474	2	8.18	0.00	8.11
497	2	25.69	0.00	3.46
535	2	34.76	0.00	14.14
578	2	10.55	0.00	5.44
635	2	8.17	0.00	6.52
684	2	34.87	0.00	5.78
746	2	25.82	0.00	3.99
876	2	20.47	0.00	7.53
879	2	35.89	0.00	14.17
883	2	0.00	1.00	1.80
950	2	23.82	0.00	12.83
962	2	10.18	0.00	7.51
981	2	36.20	0.00	11.34
D3(BJ)-B3LYP/6-31G(d,p)				
Conformation No.	Model	Relative Energy (kcal/mol)	Mol Fraction	CCS % Error
34	2	14.23	0.00	10.75
62	2	28.04	0.00	7.10
102	2	23.24	0.00	10.59
123	2	19.29	0.00	8.27
222	2	16.97	0.00	2.57
304	2	16.89	0.00	7.01
445	2	28.58	0.00	6.60
472	2	2.32	0.02	2.41
474	2	8.35	0.00	7.96
497	2	5.72	0.00	4.28
535	2	34.83	0.00	13.96
578	2	10.65	0.00	5.33
635	2	8.18	0.00	6.37

684	2	34.85	0.00	5.67
746	2	25.78	0.00	3.83
797	2	23.01	0.00	13.13
876	2	29.74	0.00	5.18
879	2	35.98	0.00	13.94
883	2	0.00	0.98	1.65
950	2	24.07	0.00	12.70
962	2	9.26	0.00	7.28
981	2	35.63	0.00	11.24

Table S34. FSSDR CCS Performance with *p*-type function

D3(0)-B3LYP/6-31G(d,p)				
Conformation No.	Model	Relative Energy (kcal/mol)	Mol Fraction	CCS % Error
90	1	21.27	0.00	9.35
112	1	24.20	0.00	14.17
121	1	20.94	0.00	16.59
156	1	40.41	0.00	20.00
217	1	39.93	0.00	14.53
234	1	28.47	0.00	12.27
304	1	22.30	0.00	12.23
399	1	0.00	1.00	2.95
420	1	24.00	0.00	14.82
472	1	36.77	0.00	12.02
474	1	21.03	0.00	9.59
549	1	55.81	0.00	24.57
641	1	55.40	0.00	21.07
669	1	36.48	0.00	17.23
684	1	27.02	0.00	11.52
797	1	15.75	0.00	7.08
876	1	11.31	0.00	5.95
879	1	33.71	0.00	16.58
883	1	37.74	0.00	12.11
962	1	13.98	0.00	4.86
D3(BJ)-B3LYP/6-31G(d,p)				
Conformation No.	Model	Relative Energy (kcal/mol)	Mol Fraction	CCS % Error
90	1	21.01	0.00	9.53
112	1	23.28	0.00	14.06
121	1	20.56	0.00	16.54
156	1	38.97	0.00	19.51
217	1	17.72	0.00	9.58
234	1	27.22	0.00	11.94
304	1	21.44	0.00	12.20
399	1	0.00	1.00	2.82
420	1	23.30	0.00	14.60
472	1	26.40	0.00	11.27

474	1	20.17	0.00	9.52
549	1	54.98	0.00	24.45
641	1	54.40	0.00	20.90
669	1	35.24	0.00	17.18
684	1	26.58	0.00	11.93
797	1	15.25	0.00	6.59
876	1	18.81	0.00	5.44
879	1	33.43	0.00	16.10
883	1	37.50	0.00	12.02
962	1	20.78	0.00	1.06

Table S35. GLVK CCS Performance with *p*-type function

D3(0)-B3LYP/6-31G(d,p)				
Conformation No.	Model	Relative Energy (kcal/mol)	Mol Fraction	CCS % Error
34	2	4.29	0.00	3.55
81	2	4.65	0.00	2.93
102	2	14.00	0.00	1.55
123	2	4.02	0.00	4.52
234	2	18.85	0.00	3.37
302	2	6.11	0.00	2.53
370	2	43.03	0.00	16.95
448	2	5.74	0.00	4.33
472	2	42.06	0.00	16.16
584	2	7.20	0.00	0.21
605	2	33.67	0.00	8.26
684	2	36.17	0.00	6.99
731	2	10.87	0.00	4.28
746	2	37.92	0.00	10.68
876	2	0.00	0.65	1.63
879	2	45.20	0.00	16.36
883	2	7.08	0.00	4.31
950	2	0.37	0.35	3.67
962	2	22.50	0.00	6.98
983	2	8.96	0.00	1.25
D3(BJ)-B3LYP/6-31G(d,p)				
Conformation No.	Model	Relative Energy (kcal/mol)	Mol Fraction	CCS % Error
34	2	11.10	0.00	3.27
81	2	11.56	0.00	2.89
102	2	21.65	0.00	1.47
123	2	6.44	0.00	3.28
234	2	0.00	1.00	2.71
302	2	10.99	0.00	3.62
370	2	50.33	0.00	16.76
448	2	15.32	0.00	4.36
472	2	49.39	0.00	16.13

584	2	11.91	0.00	0.68
605	2	40.84	0.00	8.06
684	2	42.41	0.00	8.67
731	2	20.40	0.00	3.56
746	2	45.37	0.00	10.93
876	2	5.76	0.00	2.24
879	2	15.39	0.00	4.45
883	2	16.78	0.00	2.29
950	2	7.05	0.00	3.62
962	2	13.73	0.00	4.50
983	2	15.46	0.00	1.37

Table S36. LWSAK CCS Performance with *p*-type function

D3(0)-B3LYP/6-31G(d,p)				
Conformation No.	Model	Relative Energy (kcal/mol)	Mol Fraction	CCS % Error
18	1	88.21	0.00	23.72
34	1	87.05	0.00	19.48
102	1	0.00	1.00	0.64
112	1	41.70	0.00	8.65
231	1	38.36	0.00	16.66
288	1	65.45	0.00	14.89
315	1	69.94	0.00	19.66
428	1	77.00	0.00	18.72
459	1	21.37	0.00	5.44
472	1	101.76	0.00	24.95
474	1	35.74	0.00	10.11
549	1	55.14	0.00	17.39
669	1	31.12	0.00	11.86
727	1	78.05	0.00	21.33
746	1	71.64	0.00	13.13
771	1	43.23	0.00	15.16
772	1	22.29	0.00	9.40
879	1	41.57	0.00	18.01
950	1	75.21	0.00	15.80
961	1	78.31	0.00	10.22
962	1	40.90	0.00	15.55
D3(BJ)-B3LYP/6-31G(d,p)				
Conformation No.	Model	Relative Energy (kcal/mol)	Mol Fraction	CCS % Error
18	1	65.93	0.00	23.59
34	1	65.74	0.00	19.40
102	1	9.35	0.00	9.82
112	1	36.08	0.00	7.21
231	1	16.14	0.00	14.21
288	1	11.20	0.00	2.71
315	1	47.83	0.00	19.41

428	1	48.37	0.00	16.18
459	1	0.00	1.00	5.19
472	1	19.62	0.00	19.61
474	1	14.38	0.00	10.28
549	1	34.31	0.00	17.20
669	1	9.79	0.00	11.79
727	1	20.67	0.00	18.45
746	1	46.11	0.00	13.27
771	1	22.13	0.00	14.93
772	1	1.79	0.05	9.26
879	1	17.44	0.00	17.29
950	1	37.02	0.00	14.43
961	1	36.76	0.00	7.33
962	1	18.77	0.00	15.41

Table S37. NFNR CCS Performance with *p*-type function

D3(0)-B3LYP/6-31G(d,p)				
Conformation No.	Model	Relative Energy (kcal/mol)	Mol Fraction	CCS % Error
34	2	16.11	0	6.89
102	2	18.35	0	8.93
112	2	27.88	0	16.39
123	2	32.56	0	13.12
156	2	28.37	0	13.02
433	2	10.57	0	10.26
472	2	0.00	1	2.19
549	2	22.74	0	12.37
684	2	16.58	0	9.68
727	2	27.29	0	12.26
746	2	13.41	0	4.41
772	2	26.67	0	10.39
879	2	12.49	0	9.10
883	2	16.43	0	10.48
950	2	14.91	0	9.08
962	2	6.87	0	9.43
D3(BJ)-B3LYP/6-31G(d,p)				
Conformation No.	Model	Relative Energy (kcal/mol)	Mol Fraction	CCS % Error
34	2	19.28	0	6.59
102	2	22.74	0	8.89
112	2	31.41	0	16.25
123	2	35.94	0	13.26
156	2	31.85	0	12.81
433	2	14.65	0	9.82
472	2	0.00	1	3.18
549	2	7.27	0	9.74
684	2	20.09	0	9.34

727	2	24.27	0	10.32
746	2	16.06	0	4.22
772	2	35.35	0	11.68
879	2	13.50	0	7.42
883	2	10.17	0	0.13
950	2	16.58	0	3.71
962	2	27.24	0	11.52

Table S38. NIATSGK CCS Performance with *p*-type function

D3(0)-B3LYP/6-31G(d,p)				
Conformation No.	Model	Relative Energy (kcal/mol)	Mol Fraction	CCS % Error
112	4	54.22	0.00	21.26
121	4	37.04	0.00	21.94
197	4	44.90	0.00	18.38
234	4	11.72	0.00	6.92
420	4	61.08	0.00	16.58
472	4	19.21	0.00	10.64
538	4	34.50	0.00	7.70
669	4	24.06	0.00	9.00
727	4	36.10	0.00	11.73
771	4	58.19	0.00	16.22
772	4	13.35	0.00	1.47
797	4	257.65	0.00	16.82
807	4	80.62	0.00	18.25
828	4	0.00	1.00	1.95
879	4	45.04	0.00	7.99
883	4	29.49	0.00	16.07
950	4	39.27	0.00	16.48
962	4	56.25	0.00	17.55
D3(BJ)-B3LYP/6-31G(d,p)				
Conformation No.	Model	Relative Energy (kcal/mol)	Mol Fraction	CCS % Error
112	4	64.88	0.00	20.81
121	4	41.77	0.00	21.79
197	4	50.09	0.00	18.40
234	4	20.36	0.00	4.44
420	4	36.74	0.00	5.87
472	4	24.75	0.00	11.07
538	4	19.86	0.00	3.92
669	4	12.94	0.00	6.48
727	4	28.93	0.00	9.57
771	4	68.54	0.00	17.36
772	4	19.67	0.00	3.74
797	4	134.75	0.00	14.05
807	4	71.11	0.00	16.70
828	4	0.00	1.00	0.41

879	4	52.07	0.00	7.86
883	4	31.87	0.00	16.85
950	4	44.63	0.00	17.19
962	4	69.02	0.00	16.78

Table S39. Poly-G6 CCS Performance with *p*-type function

D3(0)-B3LYP/6-31G(d,p)				
Conformation No.	Model	Relative Energy (kcal/mol)	Mol Fraction	CCS % Error
34	1	5.10	0.00	6.67
35	1	12.47	0.00	0.04
95	1	39.67	0.00	21.64
112	1	14.29	0.00	4.58
197	1	43.67	0.00	25.51
315	1	40.68	0.00	24.77
428	1	16.24	0.00	12.50
472	1	31.50	0.00	18.26
538	1	0.00	1.00	0.78
549	1	46.04	0.00	23.72
584	1	6.51	0.00	3.48
607	1	45.37	0.00	24.62
669	1	41.93	0.00	14.83
684	1	3.80	0.00	0.72
705	1	20.06	0.00	11.87
727	1	42.88	0.00	16.80
797	1	16.98	0.00	3.68
824	1	7.60	0.00	1.30
879	1	8.31	0.00	3.88
883	1	26.29	0.00	9.20
961	1	6.51	0.00	3.18
D3(BJ)-B3LYP/6-31G(d,p)				
Conformation No.	Model	Relative Energy (kcal/mol)	Mol Fraction	CCS % Error
34	1	1.08	0.11	6.31
35	1	12.48	0.00	3.01
95	1	34.79	0.00	21.67
112	1	10.53	0.00	4.50
197	1	38.46	0.00	25.29
315	1	35.41	0.00	24.65
428	1	11.76	0.00	12.39
472	1	26.90	0.00	18.13
538	1	0.71	0.21	0.21
549	1	41.17	0.00	23.76
584	1	3.12	0.00	3.34
607	1	40.36	0.00	24.33
669	1	17.28	0.00	3.25
684	1	45.82	0.00	13.37

705	1	22.42	0.00	13.50
727	1	38.16	0.00	16.73
797	1	13.43	0.00	3.89
824	1	3.63	0.00	1.52
879	1	0.00	0.68	2.00
883	1	24.52	0.00	7.87
961	1	3.12	0.00	3.03

Table S40. Poly-G8 CCS Performance with *p*-type function

D3(0)-B3LYP/6-31G(d,p)				
Conformation No.	Model	Relative Energy (kcal/mol)	Mol Fraction	CCS % Error
34	1	15.44	0.00	1.54
60	1	32.83	0.00	15.15
64	1	63.01	0.00	25.46
84	1	46.41	0.00	21.96
90	1	58.48	0.00	26.07
102	1	24.72	0.00	3.65
123	1	53.98	0.00	18.41
137	1	14.68	0.00	2.01
156	1	55.13	0.00	23.88
197	1	58.31	0.00	22.42
222	1	60.53	0.00	21.06
231	1	54.70	0.00	18.09
302	1	16.19	0.00	0.99
304	1	15.93	0.00	0.27
306	1	20.49	0.00	6.73
315	1	38.67	0.00	13.40
394	1	45.77	0.00	16.60
472	1	28.68	0.00	2.92
510	1	20.80	0.00	5.41
521	1	32.82	0.00	10.42
549	1	20.10	0.00	0.23
578	1	33.00	0.00	13.16
607	1	5.59	0.00	2.45
617	1	26.62	0.00	2.60
684	1	33.72	0.00	7.91
771	1	0.00	1.00	4.43
961	1	17.07	0.00	9.60
962	1	14.19	0.00	0.69
D3(BJ)-B3LYP/6-31G(d,p)				
Conformation No.	Model	Relative Energy (kcal/mol)	Mol Fraction	CCS % Error
34	1	37.57	0.00	12.16
60	1	31.73	0.00	15.11
64	1	61.28	0.00	25.31
84	1	44.75	0.00	21.78

90	1	51.58	0.00	24.39
102	1	24.39	0.00	3.54
123	1	52.87	0.00	18.66
137	1	0.72	0.23	4.54
156	1	53.41	0.00	23.70
197	1	57.17	0.00	22.28
222	1	30.99	0.00	8.00
231	1	53.17	0.00	18.06
302	1	60.80	0.00	21.01
304	1	13.04	0.00	0.80
306	1	19.60	0.00	6.43
315	1	38.42	0.00	13.30
394	1	44.07	0.00	17.69
472	1	30.09	0.00	2.29
474	1	25.08	0.00	1.23
510	1	20.19	0.00	5.22
521	1	31.62	0.00	10.68
549	1	19.56	0.00	0.14
578	1	32.38	0.00	13.06
607	1	15.41	0.00	1.04
617	1	26.12	0.00	1.85
684	1	33.65	0.00	7.63
771	1	0.00	0.77	4.57
961	1	21.07	0.00	9.27
962	1	5.02	0.00	3.67

Table S41. Poly-G10 CCS Performance with *p*-type function

D3(0)-B3LYP/6-31G(d,p)				
Conformation No.	Model	Relative Energy (kcal/mol)	Mol Fraction	CCS % Error
34	1	3.56	0.00	8.30
81	1	17.98	0.00	16.37
90	1	0.00	0.46	3.69
211	1	0.05	0.42	4.78
217	1	23.81	0.00	9.68
234	1	1.76	0.02	4.63
242	1	16.57	0.00	12.89
351	1	10.62	0.00	11.71
446	1	12.27	0.00	10.29
474	1	25.97	0.00	12.58
497	1	0.90	0.10	5.02
510	1	7.18	0.00	5.11
538	1	7.45	0.00	5.76
549	1	3.80	0.00	6.06
574	1	4.28	0.00	7.43
684	1	17.60	0.00	12.94
796	1	39.11	0.00	26.94

876	1	29.06	0.00	11.40
879	1	6.90	0.00	9.78
883	1	7.83	0.00	12.46
D3(BJ)-B3LYP/6-31G(d,p)				
Conformation No.	Model	Relative Energy (kcal/mol)	Mol Fraction	CCS % Error
34	1	0.00	1.00	1.50
81	1	29.10	0.00	16.10
90	1	12.40	0.00	3.28
211	1	24.73	0.00	8.20
217	1	21.53	0.00	5.63
234	1	3.81	0.00	1.67
242	1	8.66	0.00	3.69
351	1	11.20	0.00	8.10
446	1	6.94	0.00	3.91
474	1	14.32	0.00	4.04
497	1	26.99	0.00	8.54
510	1	18.65	0.00	8.88
538	1	19.32	0.00	6.52
549	1	15.42	0.00	6.12
574	1	13.60	0.00	8.00
684	1	44.12	0.00	18.56
796	1	48.74	0.00	26.59
876	1	11.37	0.00	3.12
879	1	10.77	0.00	9.63
883	1	35.25	0.00	13.61
961	1	67.79	0.00	32.55
962	1	13.78	0.00	5.85

Table S42. Poly-G14 CCS Performance with *p*-type function

D3(0)-B3LYP/6-31G(d,p)				
Conformation No.	Model	Relative Energy (kcal/mol)	Mol Fraction	CCS % Error
12	1	31.07	0	10.80
145	1	42.14	0	22.26
156	1	7.40	0	2.13
172	1	18.19	0	6.64
202	1	24.38	0	9.15
205	1	54.02	0	16.22
242	1	12.57	0	3.86
333	1	16.37	0	4.69
497	1	43.45	0	10.43
558	1	0.00	1	4.03
578	1	30.49	0	12.08
584	1	54.94	0	18.45
702	1	16.78	0	9.52
746	1	24.92	0	3.48

D3(BJ)-B3LYP/6-31G(d,p)				
Conformation No.	Model	Relative Energy (kcal/mol)	Mol Fraction	CCS % Error
12	1	33.18	0	10.96
145	1	37.62	0	22.19
156	1	0.00	1	3.65
172	1	17.61	0	10.06
202	1	36.89	0	10.12
205	1	60.60	0	17.30
242	1	12.81	0	7.33
333	1	33.56	0	9.56
497	1	31.64	0	10.18
558	1	25.22	0	5.54
578	1	26.83	0	12.25
584	1	50.30	0	18.30
702	1	41.19	0	10.59
746	1	32.79	0	13.12

Table S43. TFAEALR CCS Performance with *p*-type function

D3(0)-B3LYP/6-31G(d,p)				
Conformation No.	Model	Relative Energy (kcal/mol)	Mol Fraction	CCS % Error
1	3	25.44	0.00	16.80
2	3	10.95	0.00	14.55
3	3	22.61	0.00	9.25
4	3	21.26	0.00	17.46
5	3	25.12	0.00	14.06
6	3	20.83	0.00	13.04
7	3	22.73	0.00	14.37
8	3	25.52	0.00	15.60
9	3	17.04	0.00	12.70
10	3	31.26	0.00	11.78
11	3	29.62	0.00	13.27
12	3	14.16	0.00	7.02
13	3	36.57	0.00	17.30
14	3	55.62	0.00	23.46
15	3	25.12	0.00	14.91
16	3	24.06	0.00	4.24
17	3	0.93	0.17	8.72
18	3	5.58	0.00	1.97
19	3	39.30	0.00	22.28
20	3	0.00	0.83	5.34
D3(BJ)-B3LYP/6-31G(d,p)				
Conformation No.	Model	Relative Energy (kcal/mol)	Mol Fraction	CCS % Error
1	3	23.70	0.00	16.68
2	3	29.57	0.00	15.22

3	3	21.36	0.00	9.38
4	3	19.58	0.00	16.83
5	3	23.23	0.00	14.09
6	3	18.86	0.00	12.96
7	3	20.88	0.00	14.27
8	3	23.60	0.00	15.39
9	3	15.46	0.00	12.52
10	3	29.70	0.00	11.95
11	3	28.20	0.00	13.32
12	3	12.47	0.00	6.84
13	3	34.55	0.00	17.03
14	3	54.47	0.00	23.46
15	3	23.77	0.00	14.86
16	3	22.98	0.00	4.36
17	3	14.08	0.00	14.87
18	3	5.22	0.00	2.47
19	3	36.67	0.00	22.38
20	3	0.00	1.00	5.03

Table S44. TIAQYAR CCS Performance with *p*-type function

D3(0)-B3LYP/6-31G(d,p)				
Conformation No.	Model	Relative Energy (kcal/mol)	Mol Fraction	CCS % Error
34	1	38.99	0.00	13.57
123	1	55.20	0.00	24.88
156	1	48.63	0.00	21.50
234	1	54.08	0.00	25.08
242	1	78.24	0.00	21.38
364	1	29.40	0.00	11.34
448	1	26.52	0.00	8.67
450	1	54.86	0.00	21.62
472	1	54.58	0.00	19.69
474	1	68.30	0.00	20.09
535	1	91.81	0.00	25.04
549	1	45.87	0.00	20.72
578	1	0.00	1.00	0.12
584	1	58.66	0.00	16.30
662	1	31.46	0.00	15.49
746	1	48.12	0.00	20.51
772	1	40.03	0.00	17.70
876	1	57.40	0.00	19.21
879	1	41.56	0.00	20.66
883	1	17.07	0.00	7.13
961	1	44.38	0.00	18.59
962	1	30.32	0.00	5.30
966	1	29.43	0.00	12.69
D3(BJ)-B3LYP/6-31G(d,p)				

Conformation No.	Model	Relative Energy (kcal/mol)	Mol Fraction	CCS % Error
34	1	52.16	0.00	15.93
123	1	65.43	0.00	24.93
156	1	59.49	0.00	21.54
234	1	67.41	0.00	24.63
242	1	89.33	0.00	21.13
364	1	45.72	0.00	11.77
448	1	60.28	0.00	17.31
450	1	66.11	0.00	21.53
472	1	54.17	0.00	19.49
474	1	141.27	0.00	29.54
535	1	103.23	0.00	24.91
549	1	57.49	0.00	20.18
578	1	0.00	1.00	0.80
584	1	104.61	0.00	26.47
662	1	82.33	0.00	19.80
746	1	57.97	0.00	17.97
772	1	56.99	0.00	16.52
876	1	85.45	0.00	23.52
879	1	52.23	0.00	20.32
883	1	29.52	0.00	6.74
961	1	50.70	0.00	18.57
962	1	17.39	0.00	0.16
966	1	41.29	0.00	12.70

Table S45. VASLR CCS Performance with *p*-type function

D3(0)-B3LYP/6-31G(d,p)				
Conformation No.	Model	Relative Energy (kcal/mol)	Mol Fraction	CCS % Error
34	1	57.14	0.00	12.01
112	1	38.48	0.00	9.41
123	1	69.93	0.00	7.16
126	1	43.02	0.00	17.76
231	1	69.50	0.00	14.57
242	1	54.87	0.00	14.10
315	1	42.50	0.00	11.48
472	1	40.69	0.00	14.55
474	1	41.91	0.00	18.47
549	1	40.08	0.00	18.55
586	1	47.29	0.00	13.43
684	1	45.20	0.00	16.09
746	1	53.25	0.00	16.03
771	1	48.21	0.00	10.62
796	1	44.67	0.00	9.00
879	1	45.00	0.00	11.63
883	1	39.93	0.00	9.48

914	1	0.00	1.00	1.44
961	1	65.19	0.00	15.23
966	1	50.89	0.00	19.20
D3(BJ)-B3LYP/6-31G(d,p)				
Conformation No.	Model	Relative Energy (kcal/mol)	Mol Fraction	CCS % Error
34	1	56.44	0.00	11.28
112	1	37.05	0.00	9.40
123	1	69.36	0.00	7.11
126	1	31.87	0.00	7.79
231	1	73.04	0.00	15.17
242	1	53.85	0.00	13.91
315	1	41.82	0.00	11.40
472	1	39.51	0.00	14.47
474	1	41.76	0.00	18.09
549	1	38.55	0.00	18.42
586	1	46.38	0.00	16.85
684	1	43.63	0.00	15.84
746	1	63.35	0.00	17.00
771	1	48.20	0.00	10.29
796	1	36.54	0.00	9.84
879	1	41.67	0.00	13.11
883	1	48.04	0.00	11.98
914	1	0.00	1.00	1.62
961	1	63.63	0.00	14.80
966	1	49.35	0.00	19.04

Table S46. WIR CCS Performance with *p*-type function

D3(0)-B3LYP/6-31G(d,p)				
Conformation No.	Model	Relative Energy (kcal/mol)	Mol Fraction	CCS % Error
34	5	76.04	0.00	13.40
112	5	81.80	0.00	13.43
123	5	53.48	0.00	5.16
156	5	54.14	0.00	9.30
217	5	15.97	0.00	5.38
234	5	12.49	0.00	3.73
304	5	75.11	0.00	10.11
472	5	52.90	0.00	8.69
474	5	5.96	0.00	1.46
578	5	0.00	1.00	3.73
600	5	21.98	0.00	2.79
605	5	10.67	0.00	2.77
617	5	36.55	0.00	5.53
684	5	78.93	0.00	14.08
727	5	59.92	0.00	7.22
746	5	24.59	0.00	0.42

879	5	53.19	0.00	4.28
883	5	62.22	0.00	9.49
950	5	51.22	0.00	4.97
962	5	14.23	0.00	3.49
		D3(BJ)-B3LYP/6-31G(d,p)		
Conformation No.	Model	Relative Energy (kcal/mol)	Mol Fraction	CCS % Error
34	5	76.66	0.00	11.41
112	5	82.61	0.00	13.05
123	5	55.40	0.00	5.32
156	5	55.71	0.00	9.29
217	5	18.30	0.00	5.34
234	5	0.00	1.00	1.32
304	5	76.01	0.00	9.91
472	5	54.57	0.00	8.49
474	5	8.55	0.00	1.51
578	5	72.04	0.00	3.33
600	5	29.66	0.00	4.65
605	5	13.07	0.00	3.10
617	5	37.89	0.00	5.47
684	5	80.97	0.00	13.63
727	5	61.88	0.00	7.18
746	5	16.57	0.00	2.05
879	5	42.69	0.00	3.55
883	5	63.83	0.00	9.42
950	5	52.92	0.00	5.12
962	5	16.50	0.00	3.56
